# Witnessing eigenstates for quantum simulation of Hamiltonian spectra

**DOI:** 10.1126/sciadv.aap9646

**Published:** 2018-01-26

**Authors:** Raffaele Santagati, Jianwei Wang, Antonio A. Gentile, Stefano Paesani, Nathan Wiebe, Jarrod R. McClean, Sam Morley-Short, Peter J. Shadbolt, Damien Bonneau, Joshua W. Silverstone, David P. Tew, Xiaoqi Zhou, Jeremy L. O’Brien, Mark G. Thompson

**Affiliations:** 1 Quantum Engineering Technology Labs, H. H. Wills Physics Laboratory and Department of Electrical and Electronic Engineering, University of Bristol, Bristol BS8 1FD, UK.; 2 Quantum Architectures and Computation Group, Microsoft Research, Redmond, WA 98052, USA.; 3 Computational Research Division, Lawrence Berkeley National Laboratory, Berkeley, CA 94720, USA.; 4 Google Inc., Venice, CA 90291, USA.; 5 Quantum Engineering Centre for Doctoral Training, H. H. Wills Physics Laboratory and Department of Electrical and Electronic Engineering, University of Bristol, Bristol BS8 1FD, UK.; 6 Department of Physics, Imperial College London, London, SW7 2AZ, UK.; 7 School of Chemistry, University of Bristol, Bristol BS8 1TS, UK.; 8 Max Planck Institute for Solid State Research, Heisenbergstraße 1, 70569 Stuttgart, Germany.; 9 State Key Laboratory of Optoelectronic Materials and Technologies and School of Physics, Sun Yat-sen University, Guangzhou 510275, China.

## Abstract

The efficient calculation of Hamiltonian spectra, a problem often intractable on classical machines, can find application in many fields, from physics to chemistry. We introduce the concept of an “eigenstate witness” and, through it, provide a new quantum approach that combines variational methods and phase estimation to approximate eigenvalues for both ground and excited states. This protocol is experimentally verified on a programmable silicon quantum photonic chip, a mass-manufacturable platform, which embeds entangled state generation, arbitrary controlled unitary operations, and projective measurements. Both ground and excited states are experimentally found with fidelities >99%, and their eigenvalues are estimated with 32 bits of precision. We also investigate and discuss the scalability of the approach and study its performance through numerical simulations of more complex Hamiltonians. This result shows promising progress toward quantum chemistry on quantum computers.

## Teaser

We introduce the concept of an eigenstate witness and use it to find energies of quantum systems with quantum computers.

## INTRODUCTION

The simulation of quantum mechanical systems, using conventional classical methods, requires resources that make the problems rapidly intractable when the size of the system grows (*1*). Since Feynman’s seminal proposal, several algorithms for quantum simulation have followed (*2*–*4*), and many demonstrations have been reported in different physical systems (*5*–*14*). Calculating the spectrum of a given Hamiltonian is a fundamental problem of widespread applicability, necessary, for example, to understand reaction rates or optical spectra in quantum chemistry. In particular, characterization of excited states is required to study energy and charge transfer processes, such as those in bulk heterojunction solar cells or photosynthetic light-harvesting complexes (*5*–*16*). These kinds of problems are hard for classical computers and, in the most general case, for quantum computers as well. However, quantum devices are expected to provide scalable solutions to some instances of interest (*2*, *3*). Furthermore, also in those cases where classical methods can successfully describe the ground state (for example, for weakly interacting Hamiltonians), excited states are often hard to access (*17*, *18*), increasing the interest toward quantum methods able to address the problem of finding an efficient description of excited states. Here, we show promising progress in this direction by introducing the concept of an “eigenstate witness,” a quantity that has no known efficient analog in classical algorithms. This witness is based on the entropy acquired by a quantum register, whose time evolution is controlled by an ancillary qubit.

Given an approximate eigenstate, the quantum phase estimation algorithm (QPEA) can efficiently estimate the corresponding eigenvalue. A more practical version, the iterative phase estimation algorithm (IPEA) (*19*), has been demonstrated using different quantum hardware, such as nuclear magnetic resonance, photonic, and superconducting systems (*9*–*11*). To prepare the input eigenstates, adiabatic state preparation has been proposed as a potentially scalable solution (*3*), at the cost of expensive state preparation and deep circuits, making it unsuitable for near-term implementations on quantum computers.

Variational quantum eigensolvers (VQEs), using a hybrid quantum-classical approach, were designed to address these shortcomings (*10*, *12*, *20*–*23*). These methods prepare states described via a chosen parameterization, known as ansatz, leveraging pre-existing knowledge about the system. Different types of ansatz have been proposed for the variational search, such as the unitary coupled cluster (UCC), which is among the most promising ones to tackle quantum chemistry problems (*12*, *20*, *24*). In addition, VQE methods are believed to have unique robustness to certain errors in estimating the ground state and its eigenvalue (*10*, *25*). They are, however, quadratically less precise than QPEA, because they rely on sampling for the energy estimation in the original formulation. Crucially, variational methods could only target ground states to date.

A linear response methodology and a spectrum folding method have been proposed as possible solutions (*12*, *25*, *26*). However, although the linear response methodology maintains the low coherence time advantages of the original VQE, it requires additional sampling measurements and cannot refine approximate excited states. Instead, the folded spectrum (FS) method requires a quadratic increase in the number of terms of the effective Hamiltonian and, consequently, in the computational cost of the procedure. Thus, experimental demonstrations have been limited to ground states, despite the practical importance of excited states.

Here, by introducing the concept of an eigenstate witness, we develop a new method that also targets excited states. A crucial limitation for the solution of the eigenvalue problem is that no method for eigenstate preparation is expected to be scalable in general (*3*). It remains unanswered, whether variational methods can solve particular classes of this problem. However, it is widely conjectured that eigenstates of physically relevant Hamiltonians often can be efficiently represented within an ansatz (*3*, *4*, *12*, *20*). In these cases, we estimate that the number of applications of controlled operations required to perform our algorithm increases polynomially with the size of the system.

We demonstrate this method in a proof-of-principle experiment and test its performance via numerical simulations on higher-dimensional Hamiltonians. For the implementation of the algorithm, we developed a two-qubit quantum photonic processor on a silicon photonic platform (*27*). This device embeds the key functionalities of on-chip entangled state generation (*28*–*30*), tomography (*28*, *31*), and arbitrary controlled unitary operations ($C\hat{U}$). To perform the latter, we exploited an entanglement-based scheme (*32*, *33*).

## RESULTS

### The WAVES protocol

**Fig. 1:**
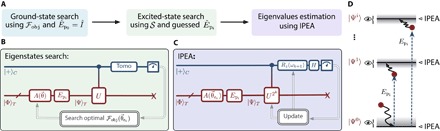
The WAVES protocol. (**A**) Flowchart describing the protocol. The optimization of $F_{\text{obj}}(\vec{\theta })=E+TS$ using the circuit in (**B**) allows one to variationally find the ground state of the Hamiltonian, preparing trial states via the ansatz $\hat{A}(\vec{\theta })$ with no perturbation $\left({\hat{E}}_{p_{0}}=\hat{I}\right)$. An initial guess for an excited state is given by a perturbation ${\hat{E}}_{p_{i}}$ on the ground state and then refined using the same circuit by exploiting the eigenstate witness $F_{\text{obj}}(\vec{\theta })=S(\text{high}‐T\;\text{limit})$. (**C**) For each target eigenstate found, the eigenvalues are precisely estimated via the IPEA using the quantum logic circuit, where *H* is the Hadamard gate. The color coding in (B) and (C), blue for the control and red for the target, refers to the difference in wavelength between the photon in the control qubit and the one in the target register in our experimental implementation. (**D**) Diagram schematically representing the intuition behind the proposed approach, where initial guesses of excited states are variationally refined using the witness and IPEA returns the eigenvalues.

The approach proposed here, witness-assisted variational eigenspectra solver (WAVES), is divided into three main steps (Fig. 1A): (i) an ansatz-based variational search for the ground state; (ii) a witness-assisted variational search for excited states, starting with an initial guess obtained from the ground-state reference as outlined below; and (iii) IPEA for the accurate energy estimate of the eigenstates found.

The quantum logic circuits for WAVES are shown in Fig. 1 (B and C). The search (Fig. 1B) proceeds by preparing trial states |Ψ〉_*T*_ in the target register, according to the ansatz, and setting the control qubit to |+〉_*C*_. The combined state |+〉_*C*_ ⊗ |Ψ〉_*T*_ is then evolved through a controlled unitary (*CÛ*) operation that embeds the unitary *Û* = *e*^−*iĤt*^ for the evolution of |Ψ〉_*T*_ according to the Hamiltonian *Ĥ*, for a time *t*. The emerging control qubit state ρ_*C*_ = Tr_*T*_(ρ) is then analyzed by single-qubit state tomography, from which it is possible to calculate the von Neumann entropy $S(\rho_{T})=S(\rho_{C})$. The entropy acts as an eigenstate witness: It is zero if the target state is an eigenstate of the Hamiltonian. In particular, for small *t*, the von Neumann entropy and the linear entropy are upper- and lower-bounded by monotonic functions of the support of |Ψ〉 in the eigenbasis of *Ĥ*; that is, they are sensible measures of such support (see section S1).

This measurement of the entropy enables us to variationally target excited states as well as the ground state. The control qubit also provides an energy estimator E = −Arg[_*T*_〈Ψ|*e*^− *iĤt*^|Ψ〉_*T*_]/*t*, evaluated using the off-diagonal elements of ρ_*C*_. The variationally optimal ground state simultaneously minimizes the entropy $S(\rho_{C})$ and the energy estimate E. Computationally, the task of finding the ground state can be interpreted as an optimization problem using a physically motivated objective function. Here, we adopt $F_{\text{obj}}=E+TS(\rho_{C})=E-T\;\text{Tr}[\rho_{C}\;\text{ln}(\rho_{C})]$, analogous in form to a free energy, where *T* is a parameter that trades off between energy optimization and entropy optimization. We call $P=\;\text{Tr}[\rho_{C}^{2}]$ the purity of the reduced density matrix of the control qubit, which is used to measure the linear entropy 1−P, here chosen as an approximation of the von Neumann entropy. We can therefore introduce the more practical objective function

$$F_{\text{obj}}(P,E)=E-TP=E-T\text{Tr}[\rho_{C}^{2}]$$ (1)

up to negligible constants. The optimization of F_obj_ also permits one to identify excited states, because they occupy local minima in the high-*T* limit for almost all evolution times *t* > 0 (section S1). Defining an initial reference state |Φ〉 (usually obtained by mean-field approximations) and the complex vector $\vec{\theta }$ as the list of parameters describing the ansatz-based state preparation $\hat{A}(\vec{\theta })$, that is, ${|\Psi \rangle }_{T}=\hat{A}(\vec{\theta })|\Phi \rangle$, our algorithm proceeds as follows:

(1) Variationally search for the state parameters ${\vec{\theta }}_{g}$ that minimize the objective function F_obj_, thus obtaining the unitary for the ground state ${\hat{A}}_{g}=\hat{A}({\vec{\theta }}_{g})$.

(2) Construct a unitary for an approximate *i*th target excited state via ${\hat{E}}_{p_{i}}\hat{A}({\vec{\theta }}_{g})$, with ${\hat{E}}_{p_{i}}$ being a system-dependent perturbation. Variationally search for the ${\vec{\theta }}_{e_{i}}$ that minimizes F_obj_ in the high-*T* limit (entropy), obtaining the unitary for the target excited state ${\hat{A}}_{e_{i}}={\hat{E}}_{p_{i}}\hat{A}({\vec{\theta }}_{e_{i}})$.

(3) Using the *Â*_*g*_ for the ground state or $\left\{{\hat{A}}_{e_{i}}\right\}$ for the excited ones in the state preparation, perform the IPEA, which further projects each state onto the closest eigenstate (*34*) and refines the energy estimate.

Here, we adopted a swarm optimization method for the experimental variational searches, where, for each iteration, F_obj_ is measured for a swarm of trial states (particles), randomly sampled from a prior distribution, and used to infer a posterior with lower F_obj_ (for more details on the optimization method, see section S2). We call each of the iterations an “epoch.” The computational complexity of using our variational method to learn eigenvalues of the Hamiltonian can be quantified by the number of controlled unitary operations performed in the simulation, which depend on the optimization method used. For the particle swarm gradient-free optimization, the computational cost of sampling from the eigenspectrum of *Ĥ* is described by Theorem 1. A version for gradient-based methods is instead reported in the “Computational cost of WAVES for gradient-based methods” section (both demonstrations can be found in section S3).

**Theorem 1.** *Let* $\hat{H}\in {\mathbb{C}}^{2^{n}\times 2^{n}}$ *be Hermitian and assume that after k* ∈ {1, …, *N*_iter_} *epochs, the state* $|\psi_{T}(k)\rangle =\sum_{i}\alpha_{i}(k)|\lambda_{i}\rangle$*, where Ĥ*|λ_*i*_〉 = λ_*i*_|λ_*i*_〉 *for *λ_*i*_ ≥ 0, *and that the sequence of sets of particles* $\left\{\Xi (k):=\{{\vec{\theta }}_{j}\}\right\}$ *satisfies* ${\text{max}}_{\vec{\varphi }\in \Xi (k)}{\left\|\vec{\varphi }-E_{\vec{\theta }\in \Xi (k)}(\vec{\theta })\right\|}_{\text{max}}\leq x_{\text{max}}$ *and* dim(Ξ(*k*)) = *N* ∀*k. Then, the number of applications of controlled e*^−*iĤt*^*, for* [0, π/(2‖*Ĥ*‖) ∍ *t* ∈ Θ(‖*Ĥ*‖^−1^)*, required for our particle swarm optimization algorithm to learn an eigenvalue within error ϵ, with a probability of at least 1/2 is in*

$$O(N_{\text{iter}}N\text{dim}(\vec{\theta })(\frac{{\left\|\hat{H}\right\|}^{2}}{{\text{min}}_{k}\sum_{i}{\left|\alpha_{i}(k)\right|}^{4}}+T^{2}){\left[\frac{\Gamma }{\delta }\right]}^{2}+\frac{1}{\epsilon })$$

*where* δ *is the maximum error in the evaluation of* F_obj_ *allowed and* $\left\{\epsilon_{\mu }^{2}(k)\right\}$ *(*$\left\{\epsilon_{\Sigma }^{4}(k)\right\}$*) is the corresponding tolerance in the (variance of the) trace of the covariance matrix of the sample mean. Finally, we define* $\Gamma :=\underset{k}{\text{max}}(x_{\text{max}}(k)/\epsilon_{\mu }(k),x_{\text{max}}^{2}(k)/\epsilon_{\Sigma }^{2}(k))$.

The above theorem implies that, in this regime, the relevant scaling parameter for iteration cost is the dimension of the parameter space. The problem of finding an appropriate ansatz is beyond the scope of this work: It is expected though to be polynomial in the number of spin orbitals for many physically relevant systems (*3*, *4*, *20*, *21*, *24*, *35*). Similarly, the number of swarm particles required (*N*) and the number of variational steps (*N*_iter_) depend on both the dimension of the relevant parameter space and prior knowledge about the solution. Because the particles are moved toward the true model as the algorithm learns, *N* is expected to scale polynomially (*36*) for problems, such as chemistry, where a good ansatz and a high degree of prior knowledge are possible. These considerations lead to the implicit scaling of the number of controlled unitary applications, which is expected to increase with the number of spin orbitals (*n*) in the system. The number of variational parameters, together with the number of swarm particles required for these specific ansätze to achieve chemical accuracy, will likely require empirical studies to be precisely estimated. Further breakdown in the cost estimates can be considered by decomposing the controlled unitary into fundamental gates using Trotter-Suzuki or linear combination–based methods (*37*), but here, we ignored these issues for simplicity. If Trotter-Suzuki methods are also taken into account for the simulation, then there is a factor of roughly *n*^5.5^ multiplied by the above costs (*38*).

Another fundamental point is how to choose the excitation operators used in the excited-state variational search. Consistent choices for the system- and state-specific perturbing unitaries ${\hat{E}}_{p_{i}}$, required to construct the excited states, can be inferred from readily computable properties of the simulated system (*25*). General many-body Hamiltonians for interacting particles decompose into $\hat{H}={\hat{H}}_{0}+\hat{V}$, where ${\hat{H}}_{0}=\sum_{i}\epsilon_{i}{\hat{a}}_{i}^{†}{\hat{A}}_{i}$ is a one-particle term and $\hat{V}$ is an interaction term. Because *Ĥ*_0_ dominates *Ĥ*, a transition from the ground state to an excited state can be approximated by the action of a sequence of single-excitation operators ${\hat{a}}_{i}^{†}{\hat{A}}_{j}$, each with a corresponding unitary ${\hat{E}}_{p}=\;\text{exp}[\pi /2({\hat{a}}_{i}^{†}{\hat{A}}_{j}-{\hat{a}}_{j}^{†}{\hat{A}}_{i})]$. If excitation operators based on the Hartree-Fock approximation do not provide sufficient accuracy, then alternative approximations can be used. Advanced methods, such as multiconfiguration self-consistent field approximations (*39*), may ultimately be needed for hard instances with substantial electron correlations. In the “Excitation operators for chemical Hamiltonians” section, we further discuss how post–Hartree-Fock methods can be used to tackle these problems through the use of natural orbitals.

### Silicon quantum photonic chip and experimental setup

**Fig. 2:**
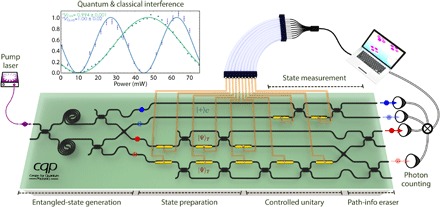
Silicon quantum photonic processor. The quantum device enables one to produce maximally path-entangled photon states, perform arbitrary single-qubit state preparation and projective measurements, and, more importantly, perform any *CÛ* operation in the two-dimensional space. Photons are guided in the silicon waveguides and controlled by thermo-optical phase shifters. Photon pairs are directly generated inside the silicon spiral sources through SFWM, off-chip–filtered and postselected by AWG filters (not shown), and measured by SNSPDs. The generated signal (blue) and idler (red) photons are different in wavelength and form the control and target qubits, respectively. The quantum chip is interfaced with a classical computer. Inset: High-visibility quantum (blue) and classical (green) interference fringes obtained in the device using the photon sources part and configuring the top final interferometer. The high visibility is essential to verify the high-performance and correct characterization of the device.

The experimental demonstration of WAVES was performed on a two-qubit silicon quantum photonic processor schematically described in Fig. 2. The device is fabricated via deep-ultraviolet lithography on a silicon-on-insulator wafer with 450 nm × 220 nm single-mode waveguides. A continuous-wave (CW) pump laser in the telecom C band with an off-chip power of approximately 10 mW is coupled into the chip using polymer spot-size converters and lensed fibers, with a facet loss of approximately 8 dB. Pairs of single photons are generated in two 1.2-cm-long waveguide spiral sources through spontaneous four-wave mixing (SFWM) (*27*). Output photons are filtered using arrayed waveguide gratings (AWGs) with a 0.9-nm bandwidth, spectrally selecting signal photons (blue) for the control qubit and idler ones (red) for the target. The photons are detected by superconducting nanowire single-photon detectors (SNSPDs) with approximately 85% efficiency, obtaining a maximum photon coincidence rate of ≈150 Hz. Optical interferometers consisting of thermo-optic phase shifters and multimode interferometer (MMI) beam-splitters are used for photonic qubit manipulation and analysis, driven by an electronic controller with 12-bit digital-to-analog converters. The automation for the WAVES algorithm, including the control of quantum gates, the data collection, and real-time analysis, is realized by a classical computer interfaced with the quantum photonic chip. More experimental details are reported in section S5.

Because of the low-power CW pump used in our experiment, multiphoton terms can be safely neglected. The use of the two spiral sources generates the Fock state $(|20\rangle +|02\rangle )/\sqrt{2}$. High-visibility two-photon quantum interference ($V_{\text{Qua}}=1.00\pm 0.02$) was observed in this experimental setup, as shown in the inset of Fig. 2. The photons are probabilistically split by two MMIs and then swapped by a waveguide crossing, yielding the maximally path-entangled state $(|1010\rangle +|0101\rangle )/\sqrt{2}$ in the Fock basis (*29*, *31*). The state of the target photon is then expanded by adding two optical spatial modes. These additional modes represent the two components of the target qubit, which is prepared in |Ψ〉_*T*_ for both paths and undergoes an arbitrary *Û*. That is, the operation performed on the target qubit—either *Î* or *Û*—depends on which path the photon is traveling on, indicated by |0〉_*P*_ or |1〉_*P*_. Path, in turn, is controlled by the state of the control qubit (the two qubits are entangled), |0〉_*C*_ or |1〉_*C*_, which yields a superposition of *Î* and *Û* gates in the circuit

$$\frac{1}{\sqrt{2}}({|0\rangle }_{C}⊗\hat{I}{|\Psi \rangle }_{T}⊗{|0\rangle }_{P}+{|1\rangle }_{C}⊗\hat{U}{|\Psi \rangle }_{T}⊗{|1\rangle }_{P})$$ (2)

By erasing the path information with the use of an additional waveguide crossing and two final MMIs and by detecting the signal photon and idler photon, we obtain the controlled unitary *CÛ* operation (*32*, *33*, *40*, *41*)

$$\frac{1}{\sqrt{2}}({|0\rangle }_{C}⊗\hat{I}{|\Psi \rangle }_{T}+{|1\rangle }_{C}⊗\hat{U}{|\Psi \rangle }_{T})$$ (3)

Note that this approach implements the *CÛ* gate without decomposing it into multiple two-qubit gates (*11*). The state preparation is realized by $\hat{A}=e^{i\varphi_{a}}e^{i\varphi_{b}{\hat{\sigma }}_{z}/2}e^{i\varphi_{c}{\hat{\sigma }}_{y}/2}$ operations, whereas the *Û* used to map the Hamiltonian is obtained by $\hat{U}=e^{i\varphi_{c}{\hat{\sigma }}_{z}/2}e^{i\varphi_{d}{\hat{\sigma }}_{y}/2}e^{i\varphi_{e}{\hat{\sigma }}_{z}/2}$, where φ_*i*_ are phases applied by on-chip thermal phase shifters. The control qubit undergoes another single-qubit operation that can be used to perform the required operations both for tomography and for the IPEA.

### Experimental results

We used the quantum photonic chip to perform WAVES, calculating the eigenspectrum of a simplified exciton transfer Hamiltonian of two chlorophyll units in the 18-mer ring of the LHII complex. We remark that this simplified model is not intended to provide an accurate description of the LHII system, yet it serves as a useful demonstration and test for our algorithm. The Hamiltonian is parameterized by α ≃ 1.46 eV, the exciton energy of a single chlorophyll unit, and β ≃ 0.037 eV, the coupling strength between the two units (*42*). This 2 × 2 Hamiltonian is written as $\hat{H}=(\alpha -\ell )\hat{I}+\beta {\hat{\sigma }}_{x}$, on the basis of Pauli operators (*20*), where *ℓ* is a reference energy that can be chosen arbitrarily (see “The single-exciton Hamiltonian: Hamiltonian parameters, mapping, and eigenvalues” section). For this Hamiltonian, the perturbing unitary for the excited state corresponds to ${\hat{E}}_{p}=e^{i\pi {\hat{\sigma }}_{z}/2}$.

**Fig. 3:**
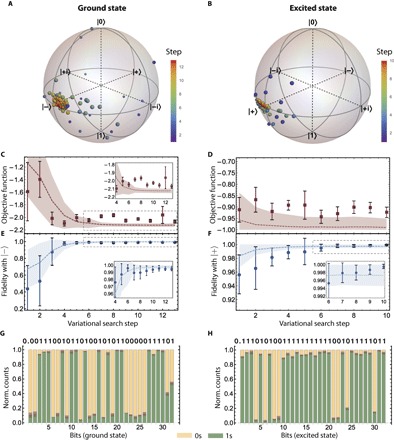
Experimental results. A Hamiltonian representing a single-exciton transfer between two chlorophyll units is implemented on the silicon quantum photonic device for an experimental test of the protocol. (**A** and **B**) Color-coded evolution of the particle swarm for the WAVES search of the ground state (| − 〉) and excited state (| + 〉) shown on the Bloch spheres. Different colors correspond to different steps of the search protocol. For the ground and the excited state searches, we report the evolution of F_obj_ in (**C**) and (**D**) and the fidelity (*F* = |〈Ψ|Ψ_ideal_〉|^2^) versus search steps in (**E**) and (**F**), converging to a final value of 99.48 ± 0.28% and 99.95 ± 0.05%, respectively. Error bars are given by the variance of the particle distribution and photon Poissonian noise. Dashed lines are numerical simulations of the performance of the algorithm, averaged over 1000 runs, with shaded areas representing a 67.5% confidence interval. Insets: Behavior close to convergence. (**G** and **H**) Normalized photon coincidences used to calculate the 32 IPEA-estimated bits of the eigenphase for both eigenstates. The theoretical bit value is shown above each bar. Errors arising from Poissonian noise are shown as shaded areas on the bars.

In Fig. 3, we show the experimental results of the WAVES approach for the ground- and excited-state search. The minimization of the objective function was performed in both cases, adopting the particle swarm method outlined above. In the experiment, the energy E and purity P used to evaluate F_obj_ were obtained by performing single-qubit tomography of the control photon. In Fig. 3 (A and B), we show the color-coded evolution of the swarm, achieving rapid convergence of the particle distribution toward the expected eigenstates of the Hamiltonian: the ground state |−〉 and the first excited state |+〉. For the ground-state search, pessimistically assuming no pre-existing knowledge of the system, the prior is initialized to uniformly span the subsection of the Hilbert space identified by the ansatz. For the excited state, instead, the search is initialized with the guessed state obtained by applying *Ê*_*p*_ to the ground state.

As shown in Fig. 3 (C and D), within 10 to 13 search steps, F_obj_ converges well to its minimal value, which corresponds to the ground state and excited state, respectively. Figure 3 (E and F) shows that the mean of the particle distribution achieves a high overlap with the eigenstate targeted: fidelities of 99.48 ± 0.28% with the ground state and 99.95 ± 0.05% with the excited state. All uncertainties are given by the variance of the prior distributions: A well-motivated error bar is among the amenable features derived from the adoption of a swarm optimization method.

The successful convergence of |Ψ〉_*T*_ is achieved by optimizing the F_obj_ function. In particular, for the ground-state search, we used a small value of *T* (*T* = 1.25) in F_obj_, whereas for the excited state case, we used $F_{\text{obj}}\equiv -P$ (that is, high-*T* limit). Imperfect measurements of F_obj_, more evident in the regime close to convergence, are mainly due to experimental noise in the phase settings, given by residual thermal cross-talk among the phase shifters. The fast convergence of the algorithm, however, indicates a good robustness of the protocol to this kind of experimental noise.

After the eigenstate search, the WAVES algorithm embeds the IPEA to improve the accuracy of eigenvalue estimates (*3*, *11*). In our implementation, we took advantage of circuit reconfigurability, mapping each ${\hat{U}}^{2^{k}}$ directly into the chip parameters (Fig. 1C). However, in universal quantum computers, *Û*^*k*^ can be efficiently achieved without classical precompilation by cascading *k* copies of *Û* (*10*). The IPEA estimated the binary fraction expansion of the eigenphase ϕ(mod 2π) for both the ground- and excited-state energies up to 32 bits (that is, a precision of 2.9 × 10^−9^ eV). The normalized photon counts are shown in Fig. 3 (G and H) for all the 32 bits. This precision is higher than what is typically achievable by spectroscopic methods.

### Numerical results for higher-dimensional systems

We complement these proof-of-principle experimental results with a set of numerical simulations, providing insight into the performance of our approach when applied to more complex Hamiltonians. For our numerical tests, we chose a set of molecular hydrogen systems (H_2_, $H_{3}^{+}$, H_3_, and H_4_). The corresponding Hamiltonians (up to 8 qubits) are represented in a Slater-type orbital basis (*43*) in the Jordan-Wigner representation (see the “Hydrogen molecules: Mapping and ansatz” section) (*20*) and exhibit several degeneracies in the spectrum. For each set of degenerate excited states, we will refer generically to the excited subspace they span.

**Fig. 4:**
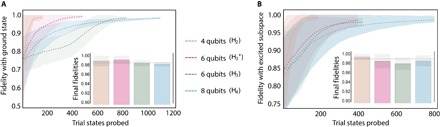
Numerical simulations for higher-dimensional Hamiltonians. The cases studied refer to molecular hydrogen systems $\left(H_{2},H_{3}^{+},H_{3},H_{4}\right)$ with the full PH ansatz. (**A**) Variational search for the ground state of each physical system. (**B**) Variational search for the targeted subspace of degenerate excited states with an initial excitation perturbation ${\hat{E}}_{p_{i}}$. On the *x* axis, we refer to the cumulative number of trial states probed (that is, the number of particles in the swarm times the variational steps). For ease of comparison, the *x*-axis origin has been shifted in (A) for the various cases to have equivalent fidelity for the average initial guess. Dashed lines denote average fidelities, with the shaded areas indicating a 67.5% confidence interval. The average fidelities achieved by the particle swarm optimization for both ground and excited states are calculated for 100 independent runs of WAVES. In all simulations, a binomial noise model has been taken into account when performing projective measurements. Insets: Bar charts summarizing final fidelities obtained by each search. All the simulations converged to the same high fidelity within errors, as indicated by the dashed black line in the inset.

Figure 4 (A and B) shows the simulation results of the ground-state search and some exemplary excited-state variational searches, respectively, addressing the latter ones with a set of excitation operators of the form ${\hat{E}}_{p_{i}}$. Note that this is only the first (variational) part of WAVES and that the second part (IPEA) will further project the state found into the eigenstate with a higher overlap. This feature is absent in previous VQE implementations. For the different cases, we increased the number of particles to 8, 16, 30, and 50 for H_2_, $H_{3}^{+}$, H_3_, and H_4_, respectively, which follows approximately linearly the number of parameters involved in the “parameterized Hamiltonian” (PH) ansatz provided in section S7 and adopted for these simulations.

In all the cases investigated here, WAVES is able to consistently find both the ground and excited states with high fidelities (≃99% in average). The insets of Fig. 4 show that the final fidelities achieved by each variational search do not decrease (within error tolerance) when increasing the size of the Hilbert space. Although these study cases do not imply scalability of the approach, they provide an encouraging result, suggesting that to keep constant the algorithm performances with the dimensionality of the problem, a subexponential increase in number of particles and iterations is enough, provided that a polynomial parameterization applies.

## DISCUSSION

We have introduced the concept of eigenstate witness and used it to develop WAVES, a new quantum method for targeting both ground and excited states of a physical Hamiltonian. We showed its proof-of-principle implementation on a silicon quantum photonic chip for a simplified exciton transfer Hamiltonian, obtaining its eigenstates with high fidelities and estimating the eigenvalues up to spectroscopic accuracy. Additional analysis of WAVES performances is provided by numerical simulations, where the protocol yields eigenstate estimates with high fidelity for Hamiltonians of up to 8 qubits.

All states found using the variational search, both in the experiments and in the numerical simulations, exhibited high fidelities with the target eigenstates. This preliminary refinement provides IPEA with an improved approximation of the target eigenstate, leading to an exponentially higher success probability in estimating the corresponding eigenvalue and reducing the overall complexity. Using IPEA, in addition to the variational search, allows the projection onto the eigenvectors, which is not guaranteed by the solely variational methods using a polynomial-sized ansatz. As the size of the system simulated increases, the shrinking of some energy gaps may lead to eigenstates close in energy being sensed as effectively degenerate by the variational witness, within the precision achievable by the experimental platform of choice. In these cases, the VQE-refined guess will exhibit consistent overlap with more than one eigenstate. Nevertheless, careful modifications to the phase estimation procedure may allow one to learn exponentially quickly either one of the eigenvalues belonging to almost-degenerate eigenstates (section S4). In summary, the variational search acts as a state preparation stage for the phase estimation, whereas the IPEA step addresses the shortcomings present in the variational ansatz.

From our study, for a particular ansatz (for example, UCC) and using low-order Trotter formulas, the time required by WAVES is expected to explicitly scale with the problem size as *O*(*Mn*^5.5^), where *n* is the number of spin orbitals and *M* is the number of variational parameters, in accordance with simulation results reported by Reiher *et al*. (*37*) for systems difficult to simulate classically, such as nitrogenase. This does not automatically imply that WAVES or any other eigenstate preparation method is efficient for any Hamiltonian, because that would imply that QMA = BQP, where QMA stands for “quantum Merlin Arthur” and BQP for “bounded-error quantum polynomial time,” respectively (*44*), which is widely believed to be false. However, an optimization based on an eigenstate witness allows the variational algorithm to address the problem of efficiently estimating an eigenvalue in the vicinity of a generic targeted state, in those cases where a polynomial-sized ansatz can be provided. This problem is generally expected to be hard on classical machines, and it is challenging to solve using traditional variational methods.

WAVES offers key improvements over previous protocols. For those instances where a good ansatz is found, it can be used to locate excited states with a quantum method in a purely variational manner, in contrast to quantum-classical linear response methods (*25*). These methods avoid the need for additional nonlinear optimization, but this may limit their accuracy, and they do not yet use quantum phase estimation to improve the final accuracy and readout precision as in WAVES. Furthermore, one can speculate how the eigenstate witness provides an independent test of the protocol’s success, detecting failure cases of convergence to local optima that do not represent a single eigenstate or excited subspace (see also section S7). These advantages come at the cost of controlling the evolution of the target register with an ancillary qubit, which is avoidable in previous VQE proposals. In addition, in WAVES, the ability to find specific eigenstates relies on the quality of the excitation operators. Further optimization on the objective function, for example, including the use of an energy penalty, can, in principle, overcome some of these limitations.

In terms of resource costs, the use of IPEA gives a quadratic speedup compared to standard VQE in estimating the energy of an eigenstate within a chosen precision. These advantages are significant given the high accuracy required in quantum chemical applications (*24*). Moreover, WAVES does not require lengthy adiabatic preparation of targeted eigenstates (*3*, *9*, *20*) or an increase in the number of terms of the implemented Hamiltonian and a precise knowledge of the spectral gap, unlike the FS method. In particular, the FS method reduces to variational optimization of a shifted and squared Hamiltonian *H*′ = (*H* − λ)^2^, thus squaring both its norm and number of terms. The choice of the shift parameter λ can also result in accidental degeneracies in the spectrum and dramatic closing of small spectral gaps. In the case of a problem, such as quantum chemistry, this can lead to *O*(*n*^8^) terms formally in the Hamiltonian, drastically increasing the cost and making it cumbersome for even small instances (*12*, *20*). Moreover, the ultimate accuracy of FS methods matches the accuracy of the variational ansatz used. However, WAVES corrects essentially all of these difficulties. It retains the original norm, spectrum, and number of terms in the Hamiltonian *O*(*n*^4^); does not depend on a shift parameter; and exceeds the accuracy of the variational ansatz used through projective phase estimation. In sections S6 and S7, the interested reader can find numerical simulations comparing WAVES with previous VQE implementations and with the FS method. The results show, in particular, that the FS method finds states with poor overlap with any true eigenstate in cases exploiting its weaknesses, whereas WAVES provides estimates exceeding 99% fidelity with a correct eigenstate in all cases tested. This direct comparison indicates higher reliability for WAVES, adding to the improvements in terms of resource costs.

In conclusion, WAVES is a new approach to tackling the search for both ground and excited states of physical Hamiltonians. The analysis performed shows that the method is expected to be scalable, under the assumption that a good ansatz can be found. The experimental demonstration on a quantum photonic chip and numerical simulations show the method performance on small-scale scenarios, indicating good noise resilience properties and better performances if compared to previous approaches. Our algorithm is, in principle, amenable to short circuit depths and leverages methods known to exhibit error robustness, thus enabling near-term experiments on non–fault-tolerant machines. By introducing new objective functions for variational algorithms, this protocol opens the way to the investigation of new methods for computing Hamiltonian spectra and represents a promising tool for future developments of quantum simulation on quantum computers.

## MATERIALS AND METHODS

### Computational cost of WAVES for gradient-based methods

In the following theorem, the computational cost for the case of gradient-based methods is reported. Proof is given in section S3.1.

**Theorem 2.** *Let* $\hat{H}\in {\mathbb{C}}^{2^{n}\times 2^{n}}$ *be Hermitian and assume that after k* ∈ {1, …, *N*_iter_} *epochs, the state* |ψ_*T*_(*k*)〉 = ∑_*i*_α_*i*_(*k*)|λ_*i*_〉*, where Ĥ*|λ_*i*_〉 = λ_*i*_|λ_*i*_〉 *for *λ_*i*_ ≥ 0*. Furthermore, assume that there exists a numerical differentiation formula that evaluates* $\partial_{\theta_{q}}F_{\text{obj}}$ *using a constant number of function evaluations on a grid of spacing h > 0 within error at most *κ(*h*Λ)^*p*^ *for positive* κ *and* Λ *for p* ∈ Θ(1)*. Then, the number of applications of controlled e*^−*iĤt*^*, for* (0, π/(2‖*Ĥ*‖) ∍ *t* ∈ Θ(‖*Ĥ*‖^−1^)*, required in the algorithm is in*

$$O(N_{\text{iter}}\kappa^{\frac{2}{p}}\Lambda^{2}\;\text{dim}(\vec{\theta })(\frac{{\left\|\hat{H}\right\|}^{2}}{{\text{min}}_{k}\sum_{i}{\left|\alpha_{i}(k)\right|}^{4}}+T^{2}){\left[\frac{\text{dim}(\vec{\theta })}{\delta }\right]}^{\frac{2p+4}{\left(p+1\right)}}+\frac{1}{\epsilon })$$

*where* δ *is the maximum error in the two-norm of the gradient of* F_obj_ *allowed and* ϵ *is the maximum error allowed in phase estimation of the final system with a probability of 1/2.*

It is then clear from the analyses contained in Theorems 1 and 2 that the particle swarm method has the potential to outperform the gradient-based method in cases where many parameters are required to describe the ansatz state and Γ is modest. However, the rate at which the two learn can differ substantially, because the same number of iterations may provide more or less information than the other case. In practice, gradient-based methods may be more practical to find an optimal solution in the vicinity of local optima, whereas global methods, such as our particle swarm method, may provide a better method for approaching them. Because the scaling of the Bayesian optimization approach with the number of variational parameters is better than the bounds that we prove for gradient-based optimization, we assumed that these approaches would be better in high-dimensional problems. Moreover, being inspired by ideas from approximate Bayesian inference, the latter retains part of their noise robustness. For these reasons, we focused on the particle swarm method for experiment and simulations in this work.

Finally, although both methods scale quadratically with *T*, in practice, the scaling will not typically be so bad. If δ is chosen to guarantee fixed relative error for the process, then the cost approaches *T*^2^/δ^2^, which is constant. This means that the quadratic scaling of *T* is not necessarily problematic in cases where the WAVES algorithm is optimizing for purity.

### Excitation operators for chemical Hamiltonians

Our method for locating excited states variationally used approximate excitation operators to enhance the rate at which excited states may be located. Quantum chemistry has a long history of using the theory of linear response to external perturbations to approximate excited states of the system (*45*). The accuracy of this approximation relies on the partitioning of the total Hamiltonian into $\hat{H}={\hat{H}}_{0}+\hat{V}$, where *Ĥ*_0_ is a noninteracting Hamiltonian of the form ${\hat{H}}_{0}=\sum_{ij}h^{ij}b_{i}^{†}b_{j}$ and $\hat{V}$ is an interacting perturbation. In quantum chemistry, this partitioning is often taken to be ${\hat{H}}_{0}=\hat{F}$, where *F* is the Fock operator that includes one-body and averaged two-body interactions, and *V* is the remainder. For many systems, $\hat{V}$ is small enough such that a perturbation treatment suffices (*46*).

As a noninteracting Hamiltonian, *Ĥ*_0_ may be efficiently diagonalized by a unitary transformation such that ${\hat{H}}_{0}=\sum_{i}\epsilon_{i}a_{i}^{†}a_{i}$, where ϵ_*i*_ are the eigenvalues of the free-fermion Hamiltonian. In this model, excited states may be formed through excitation operators of the form $a_{i}^{†}a_{j}$ acting on the ground state, where *j* indexes sites currently occupied by electrons and *i* indexes unoccupied sites. If $\hat{V}$ is comparatively small, these eigenstates will approximate eigenstates of the true Hamiltonian, and one may refine estimates within the single-particle approximation space by diagonalizing the Hamiltonian on the basis of vectors $\left\{a_{i}^{†}a_{j}|\Psi \rangle \right\}$, where |Ψ〉 is some reference state. This method is called the configuration interaction singles method. The connection may also be seen in the context of the first-order time-dependent response to an external field. For quantum computers, variations of these states may be prepared by the unitary operators ${\hat{E}}_{\text{ij}}(\theta )=\;\text{exp}[\theta (a_{i}^{†}a_{j}-a_{j}^{†}a_{i})]$.

In the case of weak interactions, classical methods, such as coupled cluster, have been successful in describing the ground state; however, even low-lying excited states in these systems may exhibit correlation structures and entanglement that prevent their efficient description. This is reflected in their difficulty of simulation by current classical methods (*17*, *18*) and represents a key motivation for quantum methods, such as WAVES, to study excited states. Moreover, we stress that the single excitations here represent initial guesses for WAVES to search through correlated states not accessible to classical simulation and that these single excitations may be derived from a reduced density matrix, using a procedure described below, which was not accessible classically due to quantum correlations in the ground state. The WAVES method refines these initial guesses through optimization and then projects to a dominant eigenstate by using phase estimation.

In quantum computing, one hopes to go beyond states that are well approximated by mean-field solutions through preparation of states with nontrivial entanglement. In the VQE approach, these states are defined by the parameterization of the ansatz; however, unlike classical approaches, we may not have efficient access to full knowledge of the wave function we are preparing. In these cases, a possible approach to generating excitation operators is to look for the “closest” one-body system. This problem defines the so-called natural orbitals in quantum chemistry (*47*), which are the orbitals that diagonalize the one-electron reduced density matrix (1-RDM) of the prepared state, given by

$$D_{ij}=\langle \Psi |a_{i}^{†}a_{j}|\Psi \rangle$$ (4)

that may be efficiently measured on any prepared quantum state, including those with entanglement. As a symmetric positive semidefinite matrix, it may be diagonalized to yield a set of excitation operators $c_{i}^{†}c_{j}$ to approximate the excited states of the interacting system. Note that in the case of an antisymmetric product state reference, such as that generated by Hartree-Fock, these orbitals are identical to those discussed above for ${\hat{H}}_{0}=\hat{F}$, as the canonical Hartree-Fock orbitals diagonalize the 1-RDM of a single antisymmetric product state.

### The single-exciton Hamiltonian: Hamiltonian parameters, mapping, and eigenvalues

Previous demonstrations of digital quantum simulation have focused almost exclusively on systems of interacting fermions such as electronic structure in molecules or the Fermi-Hubbard spin lattice model. Here, we performed numerical simulations for several such cases in section S7, reporting performances of WAVES in correctly identifying the eigenstates for the molecules H_2_ to H_4_.

However, physically interesting Hamiltonians are not restricted to interacting fermions and it is important to extend quantum simulation methodologies to general systems of interacting quantum particles and quasi-particles so that quantum simulation can have an impact on a broad range of problems relevant to physics, chemistry, biology, and materials science. The spectrum of a 2 × 2 bosonic Hamiltonian was adopted for the experimental demonstration of WAVES in the main paper. We therefore required a method to convert the bosonic Hamiltonian *e*^−*iĤt*^ into a sequence of unitary operations that can be implemented on a quantum computer. This is significant because there is not a simple analog of the Jordan-Wigner transformation that maps bosonic occupation numbers to qubits. For example, if *Ĥ* had a concise Pauli decomposition, then Trotter-Suzuki formulas can be used to write $e^{-i\hat{H}t}\approx e^{-i{\hat{P}}_{1}t}e^{-i{\hat{P}}_{2}t}\cdots$ for Pauli operators ${\hat{P}}_{1},{\hat{P}}_{2},\ldots$. General-purpose simulation methods can be used to express $\hat{H}$ as a sum of (at most) *O*(*N*^6^) one-sparse matrices, provided that *Ĥ* does not contain interactions higher than two-body (*11*, *20*). However, these methods are ill-suited for present-day experiments, because they require a coherent implementation of a graph coloring method, which requires additional qubits.

Notwithstanding this open challenge, we selected to demonstrate our WAVES approach on the exciton transfer between two chlorophyll units found in the light-harvesting complexes of purple bacteria. On the basis of localized excitons on each chlorophyll unit, the exciton transfer Hamiltonian is

$$\hat{H}=(\begin{array}{cc} \alpha & \beta \\ \beta & \alpha \end{array})$$ (5)

where α = 1.46 eV is the energy of the exciton on one of the chlorophyll units and β = 0.037 eV is the interaction between the excitons arising from the transition dipole between the two units. The qubit representation of this two-state Hamiltonian is obtained using compact mapping (*20*) and is

$${\hat{H}}_{\text{qubit}}=\alpha \hat{I}+\beta {\hat{\sigma }}^{x}$$ (6)

where $\hat{I}$ and ${\hat{\sigma }}^{x}$ are the usual Pauli matrices in the computational basis.

The WAVES approach sequentially performs a witness-assisted variational search to find the eigenstates and a QPEA to obtain an accurate energy. At this point, we recall a well-known property of eigenfunction equations: The Hamiltonian $\hat{H}\prime =\hat{H}-\ell \hat{I}$ has the same eigenstates as *Ĥ* and has eigenvalues λ′ = λ − *ℓ*, where λ are the eigenvalues of *Ĥ* and *ℓ* is a constant. The parameter *ℓ* simply redefines arbitrarily the energy zero and we were free to exploit this mathematical equivalence to improve the performance of our algorithm.

In many quantum simulation applications, the natural choice of energy zero results in Hamiltonians where the total energy is orders of magnitude larger than the energy differences relevant to the phenomena under investigation. This is particularly true, for example, for reaction energies in quantum chemistry and is also the case for our excitonic Hamiltonian where we are interested in the difference between the ground and excited state 2β ≪ α. Because QPEA requires a bitwise readout of the eigenvalue of each state of interest, any shift *ℓ* that reduces the magnitude of the corresponding energy increases the precision that can be obtained with a given length in the QPEA binary expansion. In practice, a reasonable choice for *ℓ* may be obtained, for example, from a mean-field calculation, which can be performed efficiently on a classical computer. That is, such an algorithm directly estimates the correlation energy rather than the ground-state energy. To mimic a realistic problem where mean-field theory provides a rather poor guess for the exact eigenvalues, we selected an arbitrary value of *ℓ* ≃ 1.24 eV in the experiment.

The energy estimation in WAVES adopts the form E = −Arg[_*T*_〈Ψ|*e*^−*iĤt*^|Ψ〉_*T*_]/*t*, and this imposes restrictions on the value of *t* to avoid issues due to the 2π periodicity of the Arg function. This is a limitation already known from QPEA, normally addressed by choosing *t* small enough to prevent the algorithm from providing any eigenvalues mod 2π (*11*).

However, in the WAVES protocol, additional boundaries for *t* emerge from considerations about the P estimator, as described in section S1.2. The span in purity within the accessible Hilbert space also dominated the choice of the evolution time *t* = 26. It is also easy to verify that 26(λ_*g*_ − λ_*e*_) ≠ 0 mod 2π; therefore, our choice satisfies all the conditions stated for *t*, concerning the value of P in the objective function.

### Hydrogen molecules: Mapping and ansatz

In addition to the experimental verification described, we report numerical simulations of chemical Hamiltonians using classical computers in section S7, which are partially shown in Fig. 4. In particular, we had simulated ground and excited electronic states of H_2_, $H_{3}^{+}$, and H_4_ in a STO-3G basis (*43*) in the Jordan-Wigner representation (*20*) to assess the scalability of our proposal. These represent four-, six-, and eight-qubit Hamiltonians, respectively. In our investigations, we looked at two different ansatz. First, we used a PH ansatz, where we took

$$\hat{U}(\vec{t})=\;\text{exp}[i(\sum_{ij}t_{ij}(a_{i}^{†}a_{j})+\sum_{ijkl}t_{ijkl}(a_{i}^{†}a_{j}^{†}a_{k}a_{l}))]$$ (7)

and allowed variation of the terms *t*_*ij*_ and *t*_*ijkl*_ to define the ansatz. The $a_{i}^{†}$ and *a*_*j*_ represent creation and annihilation operators in the Hartree-Fock basis, respectively. Variation was performed after transformation to Pauli operators via the Jordan-Wigner transformation. In all cases, the reference state on which $U(\vec{t})$ acts was taken to be the Hartree-Fock state with the correct number of particles. This is essentially a deformation of the original Hamiltonian, allowing one to preserve its symmetries and giving a natural connection to the original interaction structure of the problem. We also used an unrestricted UCC ansatz of the form

$$\hat{U}(\vec{t})=\;\text{exp}[\sum_{ij}t_{ij}(a_{i}^{†}a_{j}-a_{j}^{†}a_{i})+\sum_{ijkl}t_{ijkl}(a_{i}^{†}a_{j}a_{k}^{†}a_{l}-a_{l}^{†}a_{k}a_{j}^{†}a_{i})]$$ (8)

The key difference between this ansatz and the previous one is that excitations between arbitrary orbitals are allowed, not just those found in the Hamiltonian. The consequence of this is that one may create or repair symmetry-broken states that have been produced by some other means, allowing additional flexibility in the description of the state at the cost of more parameters. In the following, we will refer synthetically to a parameterization of the ansatz $\hat{A}(\vec{\theta })$, corresponding to

$$\hat{A}(\vec{\theta })=\;\text{exp}[(\sum_{i}\theta_{i}{\hat{A}}_{i})t]$$ (9)

Similarly, the approximate excitation operators used were defined in this basis as

$$E_{ij}=\;\text{exp}[\frac{\pi }{2}(a_{i}^{†}a_{j}-a_{j}^{†}a_{i})]$$ (10)

where we take *j* to index the occupied orbitals of the Hartree-Fock reference and *i* to index the occupied orbitals of the reference.

## Supplementary Material

http://advances.sciencemag.org/cgi/content/full/4/1/eaap9646/DC1

## SUPPLEMENTARY MATERIALS

Supplementary material for this article is available at http://advances.sciencemag.org/cgi/content/full/4/1/eaap9646/DC1 section S1. Notes on the objective function section S2. Swarm optimization algorithm section S3. Complexity analysis of the variational protocol section S4. Phase estimation without quantum collapse section S5. Experimental details section S6. Robustness against experimental noise of the variational search section S7. Numerical simulations of hydrogen molecules fig. S1. Median inference error in eigenvalue inference with α = 1/2 for a distribution with two randomly chosen eigenvalues and likelihood function (*37*) is used. fig. S2. Schematic representation of the experimental setup. fig. S3. Numerical simulations of the variational ground state search robustness for the bosonic one-qubit *Ĥ* against gate infidelities using F_obj_ or E alone. fig. S4. Synopsis of numerical simulations of excited states searches for the molecules H_2_ and $H_{3}^{+}$. fig. S5. Numerical simulations of the WAVES variational search for the synthetically truncated PH ansatz are studied in the molecular hydrogen systems (H_2_, $H_{3}^{+}$, H_3_, H_4_). fig. S6. Convergence of the WAVES algorithm to a subspace of excited states for different hydrogen systems. fig. S7. Comparison between different ansaetze adopted in the search for excited states in the $H_{3}^{+}$ system. fig. S8. Behavior comparison of the first part of WAVES and an equivalent implementation of the FS method when applied to the initial guess provided by the ${\hat{E}}_{p_{3}}$ excitation operator for the H_2_ system. table S1. Summary of ansätze used for simulations in the main paper and in the Supplementary Materials for the various systems investigated, along with the cardinality of their parameterization, $\text{dim}(\vec{\theta })$. table S2. Summary of possible situations occurring in numerical simulations when WAVES is performed with different ansätze and excitation operators, targeting a certain excited subspace $E_{\tau }$ from an initial guess |Ψ_0_〉. References (*48*–*56*)
