# Supplementary material for: Witnessing eigenstates for quantum simulation of Hamiltonian spectra
Source: Sci Adv. 2018 Jan 26;4(1):eaap9646. doi: 10.1126/sciadv.aap9646 (PMC5787384; doi:10.1126/sciadv.aap9646)
Supplement: http://advances.sciencemag.org/cgi/content/full/4/1/eaap9646/DC1 [file supp_4_1_eaap9646__index.html]

Science Advances | Science Advances

## Supplementary Materials

**This PDF file includes:**

- section S1. Notes on the objective function
- section S2. Swarm optimization algorithm
- section S3. Complexity analysis of the variational protocol
- section S4. Phase estimation without quantum collapse
- section S5. Experimental details
- section S6. Robustness against experimental noise of the variational search
- section S7. Numerical simulations of hydrogen molecules
- fig. S1. Median inference error in eigenvalue inference with α = 1/2 for a distribution with two randomly chosen eigenvalues and likelihood function (*37*) is used.
- fig. S2. Schematic representation of the experimental setup.
- fig. S3. Numerical simulations of the variational ground state search robustness for the bosonic one-qubit *Ĥ* against gate infidelities using *F*obj or *E* alone.
- fig. S4. Synopsis of numerical simulations of excited states searches for the molecules H2 and H+3 .
- fig. S5. Numerical simulations of the WAVES variational search for the synthetically truncated PH ansatz are studied in the molecular hydrogen systems ( H2, H+3, H3, H4).
- fig. S6. Convergence of the WAVES algorithm to a subspace of excited states for different hydrogen systems.
- fig. S7. Comparison between different ansaetze adopted in the search for excited states in the H+3 system.
- fig. S8. Behavior comparison of the first part of WAVES and an equivalent implementation of the FS method when applied to the initial guess provided by the Êp3 excitation operator for the H2 system.
- table S1. Summary of ansätze used for simulations in the main paper and in the Supplementary Materials for the various systems investigated, along with the cardinality of their parameterization, dim (θ) .
- table S2. Summary of possible situations occurring in numerical simulations when WAVES is performed with different ansätze and excitation operators, targeting a certain excited subspace *E*τ from an initial guess | Ψ0.
- References (*48–56*)

Download PDF

**Files in this Data Supplement:**

- Adobe PDF - aap9646\_SM.pdf
