# Supplementary material for: Witnessing eigenstates for quantum simulation of Hamiltonian spectra
Source: Sci Adv. 2018 Jan 26;4(1):eaap9646. doi: 10.1126/sciadv.aap9646 (PMC5787384; doi:10.1126/sciadv.aap9646)
Supplement: http://advances.sciencemag.org/cgi/content/full/4/1/eaap9646/DC1 [file aap9646_SM.pdf]

Science Advances
AAAS
advances.sciencemag.org/cgi/content/full/4/1/eaap9646/DC1

Supplementary Materials for

Witnessing eigenstates for quantum simulation of Hamiltonian spectra

Raffaele Santagati, Jianwei Wang, Antonio A. Gentile, Stefano Paesani, Nathan Wiebe, Jarrod R. McClean, Sam Morley-Short, Peter J. Shadbolt, Damien Bonneau, Joshua W. Silverstone, David P. Tew, Xiaoqi Zhou, Jeremy L. O'Brien, Mark G. Thompson

Published 26 January 2018, *Sci. Adv.* **4**, eaap9646 (2018)
DOI: 10.1126/sciadv.aap9646

This PDF file includes:

- section S1. Notes on the objective function
- section S2. Swarm optimization algorithm
- section S3. Complexity analysis of the variational protocol
- section S4. Phase estimation without quantum collapse
- section S5. Experimental details
- section S6. Robustness against experimental noise of the variational search
- section S7. Numerical simulations of hydrogen molecules
- fig. S1. Median inference error in eigenvalue inference with α = 1/2 for a distribution with two randomly chosen eigenvalues and likelihood function (*37*) is used.
- fig. S2. Schematic representation of the experimental setup.
- fig. S3. Numerical simulations of the variational ground state search robustness for the bosonic one-qubit $\hat{H}$ against gate infidelities using $\mathcal{F}_{\text{obj}}$ or $\mathcal{E}$ alone.
- fig. S4. Synopsis of numerical simulations of excited states searches for the molecules H_2 and H_3^+.
- fig. S5. Numerical simulations of the WAVES variational search for the synthetically truncated PH ansatz are studied in the molecular hydrogen systems (H_2, H_3^+, H_3, H_4).
- fig. S6. Convergence of the WAVES algorithm to a subspace of excited states for different hydrogen systems.
- fig. S7. Comparison between different ansaetze adopted in the search for excited states in the H_3^+ system.

- fig. S8. Behavior comparison of the first part of WAVES and an equivalent implementation of the FS method when applied to the initial guess provided by the $\hat{E}_{p_3}$ excitation operator for the H_2 system.
- table S1. Summary of ansätze used for simulations in the main paper and in the Supplementary Materials for the various systems investigated, along with the cardinality of their parameterization, $\dim(\vec{\theta})$.
- table S2. Summary of possible situations occurring in numerical simulations when WAVES is performed with different ansätze and excitation operators, targeting a certain excited subspace $\mathbb{E}_\tau$ from an initial guess $|\Psi_0\rangle$.
- References (*48–56*)

section S1. Notes on the objective function

In the paper, we introduced the objective function

$$\mathcal{F}_{\text{obj}} = \mathcal{E} + TS \propto b\mathcal{E} - aP \tag{1}$$

whose optimization leads the search for the eigenstates in the WAVES method. For all ground state searches, the free parameter T was fixed with an a-priori numerically optimized value (i.e. $T = 1.25$ in the 1 qubit experiment and $T = 1$ for all the simulations with $4-8$ qubits). We preferred this option, to ease the interpretation of $\mathcal{F}_{\text{obj}}$ in terms of the energy and purity witnesses.

We acknowledge that accurate a-prioristic optimization of T will not be possible in more complex cases. However, for such cases T may be left as an additional parameter to be learned during the optimization. This may be obtained running an adaptive version of the algorithm that was found optimal in noiseless simulations of the algorithm, whereas introducing a moderate amount of noise, adaptive runs tend to perform worse than optimal fixed choices for T within the same number of steps.

In the following paragraph, we consider and discuss in further detail the implications of the objective function introduced for WAVES as in SM Eq.1, showing that it has the correct properties for an eigenstate witness and justifying its adoption as an objective function. Indeed, it is not only important that $\mathcal{F}_{\text{obj}}$ achieves a local minimum at an eigenstate, but also that it behaves well in an ϵ–neighborhood about such optimal points.

We remark that multiple choices of objective functions can be investigated, here for simplicity we focus on a simple two parameters, linear objective function case.

S 1.1 Justification of Purity in the objective function

A key feature of our approach is to minimize an analogue of the free energy of the system in order to find parameters that make it as close as possible to an eigenstate prior to the phase estimation step. Here we will show that the purity of the reduced density operator of the control qubit in our method can be used to bound the support of the initial target state in the eigenbasis of the Hamiltonian.

Proof. To begin, let us assume that our variational ansatz can be written as $|\psi\rangle = \sum_j \alpha_j |\lambda_j\rangle$ such that $\hat{H}|\lambda_j\rangle = \lambda_j|\lambda_j\rangle$ for $\hat{H} \in \mathbb{C}^{2^n \times 2^n}$. Then we have that prior to the tomography step, the state can be written as

$$\frac{|0\rangle|\psi\rangle + |1\rangle e^{-i\hat{H}t}|\psi\rangle}{\sqrt{2}} \tag{2}$$

Then performing a partial trace over the second qubit register gives

$$\rho = \begin{pmatrix} \frac{1}{2} & \frac{1}{2}\left(\sum_j |\alpha_j|^2 e^{i\lambda_j t}\right) \\ \frac{1}{2}\left(\sum_j |\alpha_j|^2 e^{-i\lambda_j t}\right) & \frac{1}{2} \end{pmatrix} \tag{3}$$

Matrix multiplication then gives the following expression for the purity of the control qubit

$$\text{Tr}(\rho^2) = \frac{1}{2}\left(\sum_j |\alpha_j|^4\right) + \frac{1}{2}\left(1 + \sum_{j\neq k} |\alpha_j|^2|\alpha_k|^2 \cos([\lambda_j - \lambda_k]t)\right) \geq \sum_j |\alpha_j|^4 \tag{4}$$

This expression, closely related to the *participation ratio*, is 1 if and only if $\alpha_j = 0$ for all j such that there exists k obeying $(\lambda_j - \lambda_k)t \neq 0 \mod 2\pi$. This will generically occur for almost all $t > 0$. Thus for almost all t, obtaining a purity of 1 implies that $|\psi\rangle$ is an eigenstate of the Hamiltonian. Thus maximizing the purity is a well justified goal for our protocol. □

An even clearer result emerges if the evolution time is assumed to be short, i.e. $\max_{j,k} |\lambda_j - \lambda_k| t \leq \pi/2$, then Jensen's inequality gives

$$\text{Tr}(\rho^2) \leq \frac{\left(1 + \cos\left(\sum_{j,k} |\alpha_j|^2|\alpha_k|^2|\lambda_j - \lambda_k|t\right)\right)}{2} \tag{5}$$

the right hand side of which is a function that decreases monotonically with the average eigenvalue gap. Similarly, Eq. (4) shows that the purity is lower bounded by the participation ratio, the reciprocal of which is a frequently used estimate of the support of a state. This shows that, under the assumptions of relatively short evolution time, the purity

is upper and lower bounded by sensible measures of the support of a state, thus strengthening its adoption as a sensible measure. One can also push beyond the limitations posed by short evolutions by replacing the maximization of the purity at a fixed time with a maximization of the infinite time average of the purity.

$$\lim_{T\to\infty}\frac{1}{T}\int_0^T \mathrm{Tr}(\rho^2)\mathrm{d}t = \frac{1}{2}\left(1+\sum_j |\alpha_j|^4\right) \tag{6}$$

We see from this that maximizing the average of the purity over sufficiently long evolution times is equivalent to maximizing the participation ratio, the reciprocal of which gives the support of the eigenstate in the eigenbasis of the Hamiltonian. Thus a tight connection can be made between the time averaged purity and the support of the ansatz state in the WAVES algorithm.

S 1.2 Choice of appropriate t for the experimental implementation

We are interested in choosing an evolution time t, such that we obtain a lower bound far from 1 in Supp. Eq. 4, for the purity $\mathcal{P}$ spanned by all possible trial states. This is important for WAVES protocol, considering that eigenstate searches are (jointly) driven by $\mathcal{P}$. The global range of purity values will thus depend upon $\Lambda_M t = \max_{j,k}|\lambda_j - \lambda_k|t$, whereas the minimum purity observed when searching the Hilbert space between two generic eigenstates will depend upon their energy difference $\Lambda_M t = \min_{j,k}|\lambda_j - \lambda_k|t$, i.e. on the degeneracy of the eigenspectrum.
If purity were to span a small range of values in the Hilbert subspace searched, a higher accuracy in the measurements would be required in turn, in order to make eigenstate witnesses sufficiently robust against experimental noise. This could be achieved, for example, via an increase in the number of measurements to obtain each data-point. Therefore, t shall be chosen big enough to keep the number of measurements as small as possible. At the same time, it is important to satisfy the requirement to invoke Jensen's inequality for $\mathcal{P}$ to be a sensible measure of the eigenstates' support.
Experimentally, therefore, a meaningful choice is to increase t to make it as close as possible to the aforementioned upper bound $\Lambda_M t \leq \pi/2$, in order to reduce the accuracy required for $\mathcal{P}$ measurements. This can be achieved using e.g. mean field estimates of the eigenvalues λ_j we are interested in. If for such choice $\Lambda_M t \ll \pi/2$, then either a corresponding increase in the number of measurements, or a different witness as in Supp. Eq. 6 may be necessary.

S 1.3 Von Neumann entropy

After having discussed the scaling of the purity, we will now focus on showing that, for small t, the von Neumann entropy of the control qubit is also a well motivated measure of the support of the state. From the fact that the linear entropy is a lower bound on the von Neumann entropy we have that if $\max_{j,k}|\lambda_j - \lambda_k|t \leq \pi/2$ then

$$\mathrm{Tr}(-\rho\log(\rho)) \geq 1 - \mathrm{Tr}(\rho^2) \geq \frac{\left(1-\cos\left(\sum_{j,k}|\alpha_j|^2|\alpha_k|^2|\lambda_j-\lambda_k|t\right)\right)}{2} \tag{7}$$

Thus the von Neumann entropy is bounded below by a monotonically increasing function in the average eigenvalue gap under our assumptions on t. We similarly see from (7) and (6) that the lower bound holds for almost all t.

We can show a similar upper bound through the use of Jensen's inequality

$$\mathrm{Tr}(-\rho\log(\rho)) \leq -\log\left(\frac{1}{2}+\frac{1}{2}\left(\sum_{j,k}|\alpha_j|^2|\alpha_k|^2\cos([\lambda_j-\lambda_k]t)\right)\right) \tag{8}$$

If we now assume that $\max_{j,k}|\lambda_j-\lambda_k|t < 2$ then we see from the bound $\cos(x) \geq 1 - x^2/2$ and the monotonicity of $\log(x)$ that

$$\mathrm{Tr}(-\rho\log(\rho)) \leq -\log\left(1-\frac{1}{4}\left(\sum_{j,k}|\alpha_j|^2|\alpha_k|^2[\lambda_j-\lambda_k]^2t^2\right)\right) \tag{9}$$

This shows that the von Neumann entropy is also bounded above by a monotonically increasing function of the expected square of the gap, therefore it is a well motivated measure of the support of the state in the eigenbasis of $\hat{H}$.

section S2. Swarm optimization algorithm

We discuss here the optimization algorithm employed for the minimization of $\mathcal{F}_{\text{obj}}$ in our experiment, which is a gradient–free particle swarm optimization algorithm. Other optimization methods may be adopted for this subroutine of the protocol, however this swarm approach is inspired by ideas from approximate Bayesian inference, thus retaining part of their noise robustness. The idea is to have a swarm of trial states (particles) randomly sampled from a prior distribution. $\mathcal{F}_{\text{obj}}$ is measured for each particle, and the result is used to update the probability distribution. A new swarm is drawn from the updated distribution to perform further steps, until a convergence criterion is triggered. The last inferred posterior provides the estimate for the optimal state and the corresponding objective function.

We present here the details of the procedure, whose pseudo-code is in Algorithm 1. In the case of a ground state search, we start sampling a swarm Ξ composed of N particles (i.e. a set of N parameters values $\{\vec{\theta}_i\}$) from the parameter space according to a uniform distribution (we assume no prior knowledge of the ground state). For each of the particles, the quantum device can efficiently evaluate the two quantities $\mathcal{P}$ and $\mathcal{E}$, thus the objective function $\mathcal{F}_{\text{obj}}$ can be easily computed. In the pseudo-code, to emphasize operations performed within the quantum device, we prepend a "(Q)" to any step where such operations are invoked. Once the objective function has been obtained for all the particles, the algorithm rejects some of them ($N-S$ in our example), on the basis of the corresponding $\mathcal{F}_{\text{obj}}$, reducing the set to Ξ'. This subset is used to compute classically the weighted mean $\mu_{\theta'}$ and standard deviation $\sigma_{\theta'}$ of a Gaussian distribution on the parameters space. The weights of the particles are here directly proportional to their measured $\mathcal{F}_{\text{obj}}(\vec{\theta}_i)$. At each step we re-sample the swarm: $N-S$ new particles are drawn from the (last updated) Gaussian distribution $\mathcal{N}(\mu_{\theta'}, \sigma_{\theta'})$, and replace the ones filtered out in the new input set for the following step. In practice we take $S = \lceil\sqrt{N}\rceil$, but acknowledge that for more challenging examples $S \in \mathcal{O}(N)$ will likely be required.

Algorithm 1: Swarm optimization algorithm

Data: Random set of N particles, characterized by the set of parameters $\Xi := \{\vec{\theta}_i\}$, sampled uniformly from the parameters' space
initialization of parameters: a, b, *threshold(s)*, S;
while *convergence **not** achieved* **do**
  foreach $\vec{\theta}_i \in \Xi$ **do**
    (Q) evaluate control qubit *purity* ($\mathcal{P}$);
    (Q) evaluate *energy estimator* ($\mathcal{E}$);
    calculate $\mathcal{F}_{\text{obj}}(\theta') = -a\mathcal{P} + b\mathcal{E}$;
  end
  calculate $\mu'_{Fobj} = \langle \mathcal{F}_{\text{obj}}(\vec{\theta}_i)\rangle_{\Xi}$;
  if $|\mu'_{Fobj} - \mu_{Fobj}| <$ *threshold for objective function* **then**
    convergence achieved (via $\mathcal{F}_{\text{obj}}$) ;
  else
    $\mu_{Fobj} \leftarrow \mu'_{Fobj}$;
  end
  find the subset $\Xi' := \{\vec{\theta}'_i\} \subset \{\vec{\theta}_i\}$ of the S particles with lowest $\mathcal{F}_{\text{obj}}$;
  if *$\mathcal{F}_{obj}$ is adaptive* **then**
    adjust $a \propto |\langle\mathcal{P}\rangle_{\Xi'} - \langle\mathcal{P}\rangle_{\Xi}|$ and $b \propto |\langle\mathcal{E}\rangle_{\Xi'} - \langle\mathcal{E}\rangle_{\Xi}|$;
    normalize $\{a, b\}$;
  end
  calculate (weighted) mean $\mu_{\theta'} := \langle\vec{\theta}'_i\rangle_{\Xi'}$ for the Θ' subset parameters;
  calculate (weighted) variance $\sigma^2_{\theta'} := \langle(\vec{\theta}'_i)^2\rangle_{\Xi'} - \langle\vec{\theta}'_i\rangle^2_{\Xi'}$ for the Ξ' subset parameters;
  if $\sigma_{\theta'} <$ *threshold for particle dispersion* **then**
    convergence achieved (via $\sigma_{\theta'}$) ;
  end
  generate a set Ω of $N-S$ new particles, Gaussian distributed around $\mu_{\Xi'}$ with standard deviation $\hat{\sigma}_{\Xi'}$;
  $\Xi \leftarrow \Xi' \cup \Omega$;
end
Result: $\mu_{\theta'}$ is the output best estimate for the optimal parameters, and $\sigma_{\theta'}$ the error estimate.

In our implementation, convergence is achieved and the algorithm stopped either when the Gaussian distribution

is sufficiently peaked (i.e. the spread of the distribution is lower than an accuracy determined a-priori to be satisfactory for identifying the ground state), either when fluctuations in $\mathcal{F}_{\text{obj}}$ along the last steps are smaller than a pre-determined threshold (indicating that the algorithm is drawing new particles in a plateau of the objective function, and therefore a restart is recommended).

The swarm optimization in the case of excited state searches is fully equivalent, the only differences being: i) a choice of $a \gg b$ (or equivalently $T \gg 1$), ii) the initial distribution of the swarm Ξ assumed as a Gaussian distribution, centered around the initial guess $|\Psi_0\rangle = \hat{E}_{p_i}\hat{A}_g|\Phi\rangle$, as provided by the appropriate excitation operator. Several modifications in the *swarm optimization* algorithm were investigated. One possible improvement is to adaptively choose at each algorithm step the parameters $\{a, b\}$ concurring to the definition of $\mathcal{F}_{\text{obj}} = b\mathcal{E} - a\mathcal{P}$. This can be useful for non-convex optimizations or when poor a-priori information is available about values spanned by $\mathcal{E}$ and $\mathcal{P}$. A different strategy is to enhance the algorithm rapidity to converge, introducing some "greediness". This amounts to replace $\mu_{\theta'}$ with the parameters of the particle exhibiting the best $\mathcal{F}_{\text{obj}}$, $\vec{\theta'}_{best}$.

S 2.1 Optimizing the number of particles for the experimental study case

Preliminary simulations were performed in order to test the algorithm behavior for the study-case experimentally addressed in the paper. In particular, it was observed how an initial set of as few as $N = 8, S = 2$ particles was capable converging to the target state with average fidelity $F_{|-\rangle} > 0.997$, also when including a moderate level of Poissonian noise to the simulated photon counts at the output of the circuit (replacing the exact photon coincidences at the output ports of the simulated circuit, with Poissonian-distributed coincidences having a mean coincident with the numerically computed value, and a variance that assumes pessimistically a peak of ~ 200 experimental coincidence counts).

$N = 8$ was thus chosen as a reasonable trade-off between the final fidelity obtained and the total number of tomographies required, bearing in mind that in a quantum simulation framework, an overlap of $0.9 - 0.99$ with the ground state is already considered a very good starting point for a fine-grain energy estimation using IPEA *(3)*.

section S3. Complexity analysis of the variational protocol

In the article we discuss some of the main aspects concerning the computational cost of WAVES. In particular, in Theorem 1 from the Results section and Theorem 2 reported in the Methods we evaluated the computational costs neglecting the contribution due to the unitary decomposition. In the following, we report the proofs of those theorems and we further expand the discussion on the efficiency and scalability of this approach. In SM S 3.4 we also highlight some fundamental differences between WAVES and traditional VQE and we present Theorem 1, which shows how a fault tolerant quantum computer can give a quadratic improvement in such scaling.

S 3.1 Complexity of WAVES for gradient-based optimization methods: proof of Theorem 2

We will here delve into the proof of Theorem 2 in Methods, which states the complexity of gradient based simulation. A major caveat must be born in mind for any such proof. If we wish to fully bound the complexity of any variational algorithm, we need to know how many steps it will take to converge to an acceptable variational ansatz. In general, no such bound exists. So while the following bounds on the complexity will invariably contain terms for the number of steps involved in the variational optimization, it is very difficult a-priori to estimate such parameter, which depends upon the quality of the initial guess, as well as the ansatz used. In practice, a small number of variational steps was sufficient for small variational quantum eigensolvers (for example fewer than 60 are required in *(12)*), and we found that less than 70 steps were enough for all the simulations performed in SM S 7 up to 8 qubits.

Proof of Theorem 2. There are two phases of the algorithm. The first step involves variational optimization of the trial state $|\psi_T\rangle$ and the second involves using phase estimation to learn the energy accurately. The cost of the second phase is trivially $O(1/\epsilon)$ with ϵ the targeted accuracy, so we only need to focus on the former cost in this proof.

Using this objective function, we have two intermediate errors that contribute to δ. Let us define δ_1 to be the error in the gradient that arises from inexact gradient estimation and let δ_2 be the error that arises from inexact entropy calculations. Then using p^{th}-order finite difference formulas on a uniform mesh of points with side width h, the error in a single gradient evaluation is, for positive κ, Λ

$$O([\delta_1 + \delta_2]/h + \kappa(\Lambda h)^p) \tag{10}$$

The error is asymptotically optimal when both terms are equal to each other which occurs, assuming $p \in \Theta(1)$, for

$$h \in \Theta\left(\frac{\left(\frac{[\delta_1+\delta_2]\Lambda}{\kappa}\right)^{1/p+1}}{\Lambda}\right) \tag{11}$$

If we demand $\delta_1 = \delta_2$ and that the total error in the derivative estimate is δ_0 then we have from (11) that it suffices to choose

$$\delta_1 \in \Theta\left(\frac{\delta_0^{1+1/p+1}}{\kappa^{1/p}\Lambda}\right) \tag{12}$$

Now, to estimate $T\mathrm{Tr}(\rho^2)$ within error δ_1, we need to perform $O((T/\delta_1)^2)$ experiments of the form of Fig. 1.b in the main body. Supp. Eq. (12) then implies that the total number of controlled e^{-iHt} needed to perform the operation is

$$O\left(\frac{\kappa^{2/p}\Lambda^2 T^2}{\delta_0^{2+2/p+1}}\right) \tag{13}$$

Consider now the number of controlled evolutions required to learn $\mathcal{E}$ within error at most δ_1. In order to estimate this we use that if $\|\hat{H}\|t < \pi/2$ then

$$\mathcal{E} = -\tan^{-1}(y/z)/t \tag{14}$$

where $y = \mathrm{Im}(\langle\psi_T(k)|e^{-i\hat{H}t}|\psi_T(k)\rangle)$ and $\mathrm{Re}(\langle\psi_T(k)|e^{-i\hat{H}t}|\psi_T(k)\rangle)$. It is then easy to see from differentiation and standard norm inequalities that if the errors in both quantities are at most δ_3 then the total error in $\mathcal{E}$ is at most

$$\frac{\delta_1(|z|+|y|)}{t(z^2+y^2)} \leq \frac{\delta_3}{t\sqrt{z^2+y^2}} \in O\left(\frac{\delta_3\|\hat{H}\|}{\sqrt{z^2+y^2}}\right) \tag{15}$$

Now since $|\psi_T(k)\rangle := \sum_j \alpha_j(k)|\lambda_j\rangle$ and since $\pi/2 \geq \lambda_j \geq 0$ we have that

$$z^2 = (\sum_j |\alpha_j(k)|^2 \cos(\lambda_j t))^2 \geq \min_k \sum_j |\alpha_j(k)|^4 \cos^2(\lambda_j t) \tag{16}$$

and

$$y^2 = (\sum_j |\alpha_j(k)|^2 \sin(\lambda_j t))^2 \geq \min_k \sum_j |\alpha_j(k)|^4 \sin^2(\lambda_j t) \tag{17}$$

These results then imply that under our assumptions

$$z^2 + y^2 \geq \min_k \sum_j |\alpha_j(k)|^4 \tag{18}$$

Substituting this into (15) gives that the error in the evaluation of $\mathcal{E}$, δ_1, satisfies

$$\delta_1 \in O\left(\frac{\delta_3\|\hat{H}\|}{\sqrt{\min_k \sum_j |\alpha_j(k)|^4}}\right) \tag{19}$$

Therefore (12) implies

$$\delta_3 \in \Omega\left(\frac{\sqrt{\min_k \sum_j |\alpha_j(k)|^4}\delta_0^{1+1/p+1}}{\|\hat{H}\|\kappa^{1/p}\Lambda}\right) \tag{20}$$

The number of experiments needed to achieve this is $O(1/\delta_3)$, and combining this with the result in (13) shows that the number of controlled evolutions needed to learn the derivative within error δ is at most

$$O\left(\kappa^{2/p}\Lambda^2\left(\frac{\|\hat{H}\|^2}{\min_k \sum_j |\alpha_j(k)|^4} + T^2\right)\left[\frac{1}{\delta}\right]^{2+2/(p+1)}\right) \tag{21}$$

In order to compute the gradient, we need to repeat this algorithm $\dim(\vec{\theta})$ times. Furthermore, in order to ensure that the error is at most δ, with respect to the 2–norm, in the gradient of the objective function we need to compute each component of the gradient within error at most $\delta/\dim(\vec{\theta})$. Then since the gradient calculation must be repeated at most N_{iter} times the total cost of computing the gradients is in

$$O\left(N_{\mathrm{iter}}\kappa^{2/p}\Lambda^2\dim(\vec{\theta})\left(\frac{\|\hat{H}\|^2}{\min_k \sum_j |\alpha_j(k)|^4} + T^2\right)\left[\frac{\dim(\vec{\theta})}{\delta}\right]^{2+2/(p+1)}\right) \tag{22}$$

The final cost estimate then follows by adding the $O(1/\epsilon)$ cost of phase estimation to 22. □

S 3.2 Complexity of WAVES for gradient-free optimization methods: proof of Theorem 1

Now that we have discussed the cost of performing the optimization using a traditional gradient descent method, we here prove the cost of our gradient-free method below, outlined in the "Results" section of the main manuscript. Also it should be noted that in the following discussion we require that the error is small in the trace of the sample covariance matrix and the one-norm of the vector of means. In practice, such algorithms can typically tolerate small error in each of the variances and the max-norm of the vector of means. Nevertheless for the formal proof we adopted a more conservative metric, because if the norm is bounded with respect to the trace and the one-norm, then we can exclude pathologic behavior in the asymptotic limit of many-parameter problems.

Proof of Theorem 1.

The proof proceeds similarly to Theorem 2 in SM S 3.1. Again, the final phase estimation in WAVES requires $O(1/\epsilon)$ applications of the controlled unitary operation. So it suffices to focus on the variational search cost. The optimization process requires $O(NN_{iter})$ evaluations of the objective function. If we wish to learn the objective function within error δ then the cost of this process is from (19) and the discussion following (12) at most

$$O\left(N_{\text{iter}}N\left(\frac{\|\hat{H}\|^2}{\min_k \sum_j |\alpha_j(k)|^4}+T^2\right)\frac{1}{\delta^2}\right) \tag{23}$$

The next step is to constrain N such that the bounds on the variance in the sample mean of the S particles accepted by the algorithm and variance of the sample variance are appropriately bounded.

Owing to the fact that the statistical errors in the sample variance are i.d., we can use the fact that variance is additive. Thus the variance in each of the $\dim(\vec{\theta})$ components of the particles is

$$\frac{\sigma^2}{S} \le \frac{x_{\max}^2(k)}{S} \tag{24}$$

Thus if we are to guarantee that the trace of the covariance matrix of the sample mean at most $\epsilon_\mu^2(k)$ it suffices to take

$$S = \Theta\left(\dim(\vec{\theta})\max_k \frac{x_{\max}^2(k)}{\epsilon_\mu^2(k)}\right) \tag{25}$$

Similarly the variance in the sample variance in one of the components of $\vec{\theta}$ is (in the asymptotic limit $S \gg 1$)

$$\frac{\mu_4}{S}+\frac{(S-3)\sigma^2}{S(S-1)} \in O\left(\frac{x_{\max}^4(k)}{S}\right) \tag{26}$$

where μ_4 is the fourth moment of one of the components. If we then require that the variance of the trace of the covariance matrix is at most $\epsilon_\Sigma^4(k)$ then it suffices to take

$$S \in \Theta\left(\dim(\vec{\theta})\max_k\left(\frac{x_{\max}^4(k)}{\epsilon_\Sigma^4(k)}\right)\right) \tag{27}$$

It therefore suffices to take $S \in \Theta(\dim(\vec{\theta})\Gamma^2)$ Then by assumption we have that $N \in O(S)$ and hence the result

$$O\left(N_{\text{iter}}N\dim(\vec{\theta})\left(\frac{\|\hat{H}\|^2}{\min_k \sum_j |\alpha_j(k)|^4}+T^2\right)\left[\frac{\Gamma}{\delta}\right]^2+\frac{1}{\epsilon}\right) \tag{28}$$

for the overall method complexity follows from (23). □

S 3.3 Efficiency

A priori, provided that the states that emerge as part of VQE have substantial support on a small number of eigenstates, the norm of the Hamiltonian is bounded, constant precision is required and the number of variational parameters is polynomially large; WAVES is efficient given that e^{-iHt} is efficiently simulatable. First, let us focus on a particular ansatz for the state. Consider unitary coupled cluster expansions which take

$$|\psi_{UCC}\rangle = e^{T-T^\dagger}|\psi_{HF}\rangle \tag{29}$$

where $|\psi_{HF}\rangle$ is the Hartree–Fock state and $T = T_1 + T_2 + \cdots$ is the cluster operator *(12)*. Here the terms T_1 consist of single excitations away from the reference state, T_2 consists of double excitations etc. Let us restrict our interest to

$T \approx T_1 + T_2$. In this case there are, for systems of n spin orbitals, $\dim(\vec{\theta}) \in O(n^4)$ different parameters in the ansatz. In practice far fewer terms are required in the ansatz than this *(12,24)*. Also, for constant $\|\hat{H}\|$, in practice, the norm scales not with the number of spin orbitals but with the molecular properties of the system *(48)*.

In order to make a statement about the gate efficiency of the above methods we need to further consider a simulation algorithm for $e^{-i\hat{H}t}$. There are many quantum simulation algorithms that can be used to perform such a simulation efficiently (for constant error tolerance). The simplest are Trotter–decompositions, which for a system of n spin orbitals require at most $n^{8+o(1)}$ *(49)* primitive exponential operations (which can each be implemented using a logarithmic number of T gates in a Clifford $+T$ gate library *(50)*) for a second quantized Hamiltonian. Empirically, low order Trotter-decompositions are found to require a number of sequential exponentials that scale roughly as $O(n^{5.5})$. Recent work in truncated Taylor series simulations have demonstrated upper bounds on the cost that scale as $O(n^5)$ *(51)*. In all such cases, arbitrarily large molecules can be simulated at cost that scales polynomially with the number of spin orbitals in the problem.

From Theorem 1, the dominant cost of the algorithm comes from the variational steps involved (assuming ϵ and δ are fixed). Since $\|\hat{H}\|$ depends only indirectly on the number of spin orbitals (n), as are N_{iter}, Γ and N, we will treat these parameters as independent since they have no explicit dependence on the dimension. Indeed, most are simply user selected parameters. In general the dependence or not of these parameters from the size of the system needs to be verified and it is one of the main open questions concerning VQE methods. While the quantity $\min_k \sum_j |\alpha_j(k)|^4$ does depend on n, this dependence only comes in implicitly through the quality of the initial ansatz. While many ansatz-based solutions, such as Hartree-Fock, CISD or UCC states, can perform well in practice *(39)* it is challenging to estimate a priori how it will scale for a particular molecule, so we assume that it is constant in our subsequent discussion. In other words, we assume that the wave function has strong support over a constant number of eigenvectors. Under these assumptions the cost of the algorithm is then simply proportional to the cost of the simulation required for the ansatz state and the phase estimation circuit.

More specifically, the cost under the assumption of constant ϵ and δ the cost is then given from Theorem 1 to be

$$O\left(N_{\text{iter}} N \dim(\vec{\theta}) \left(\frac{\|\hat{H}\|^2}{\min_k \sum_j |\alpha_j(k)|^4} + T^2\right) \Gamma^2 (n^{5.5} + \dim(\vec{\theta})^{2.5}) + \frac{1}{\epsilon}\right) \tag{30}$$

This shows that WAVES can be used to provide a scalable method for drawing samples that are biased towards a particular eigenstate. Furthermore, if the eigenstate is in a degenerate (or near-degenerate) manifold of eigenstates, then the algorithm will learn an eigenvalue but the variational parameters may not correspond to an eigenstate when such degeneracies are broken. In this way, our algorithm provides scalable estimates of the energy even if the gap vanishes. However, we emphasize that without additional assumptions we cannot prove that any specific class of chemical systems can be efficiently estimated. We discuss such caveats in greater detail below.

S 3.4 Scalability of VQE

A major criticism levied at VQE relative to IPEA based simulation is on its scalability. To some extent neither VQE, Waves, nor any other groundstate preparation method can be scalable in general. The reason for this is that if VQE were capable of preparing a close surrogate to an arbitrary groundstate in polynomial time then it would ensure that $\mathsf{NP} \subseteq \mathsf{BQP}$ *(44)*, which is widely believed to be false under reasonable complexity theoretic assumptions. However, the remarkable success that post Hartree–Fock methods have shown in estimating groundstate energies of molecules *(39)* has lead to the conjecture that physically meaningful molecules do not have groundstates that are hard to prepare *(52)*. Furthermore, such criticisms do not apply only to VQE. They also apply to IPEA and classical approaches as well.

Although resolving the question of whether VQE can be used to scalably solve all electronic structure problems would constitute a substantial breakthrough in computational complexity theory, the question of whether the algorithm itself is scalable can be addressed. That is to say, "what is the computational complexity of implementing the VQE algorithm without requiring rigorous guarantees of the distance between the estimated energy and the true ground state energy?" Here we discuss this point and show that the algorithm is in principle scalable, and show that, through the use of quantum amplitude estimation, VQE can be made much more scalable on a fault tolerant quantum computer than it would appear from prior analyses.

WAVES consists of two phases. The first involves optimization of the training objective function. These costs are estimated in Theorem 1 and Theorem 2 which reduce to VQE when $T = 0$. Broadly speaking, provided that the state has a significant overlap with the groundstate, constant precision is required for the optimization and the number of variational parameters used scales polynomially with the number of spin-orbitals in the molecule then both methods can be applied for fixed number of iterations. In this sense the methods are efficient and scalable, although we cannot make promises apriori about whether the resultant estimates will be accurate.

The part of VQE that remains to be estimated is the number of samples needed if iterative phase estimation is not used. Since the above theorems assume the use of iterative phase estimation the costs cited do not reflect that of traditional VQE. Avoiding phase estimation does not make VQE inefficient (for constant ϵ) but it can under some circumstances render it impractical. Previous work by Wecker et al *(24)* highlights this, wherein they show that VQE requires a number of measurements to estimate the ground state energy that scales as

$$M \in O\left(\frac{\left[\sum_j |h_j|\right]^2}{\epsilon^2}\right) \tag{31}$$

given $H = \sum_j h_j H_j$ for $h_j \in \mathbb{R}$ and Hermitian H_j and ϵ is the desired error target. This to no small extent justifies using phase estimation as the final step of the algorithm.

However, in principle quadratically better scalings can be achieved using a fault tolerant quantum computer. We state this below in the following theorem.

Supp. Theorem 1. *Let $H = \sum_j h_j H_j$ for $h_j \in \mathbb{R}$ and H_j both unitary and Hermitian and let $|\psi\rangle = U|0\rangle$ for unitary U. Then $\langle\psi|H|\psi\rangle$ can be estimated to within error ϵ with high probability using $O(\sum_j |h_j|/\epsilon)$ applications of U and $U^\dagger$.*

Proof. Our algorithm proceeds as follows. For each term in the variational ansatz we use a form of amplitude estimation to estimate $\langle\psi|H_j|\psi\rangle$ within error $\epsilon/|h_i|$. We then sum the errors from these estimates to find the final answer.

These expectation values can be estimated using the Hadamard test, which yields a qubit that is 0 with probability $\mathrm{Re}\langle\psi|H_j|\psi\rangle$ or $\mathrm{Im}\langle\psi|H_j|\psi\rangle$ depending on which version of the test is used. Let V be the unitary that implements the Hadamard test (which is unitary because H_j is unitary), then we can define the following operators $I - 2V|0\rangle|\psi\rangle\langle\psi|\langle 0|V^\dagger$ and $I - 2P_0$ where P_0 is the projector onto the subspace where the qubit in question is 0. Because $|\psi\rangle = U|0\rangle$ these operators can be implemented using multiply-controlled Z gates and at most one query to U and another to $U^\dagger$. Since the Grover oracle is found by concatenating these two operators, it can be implemented using $\Theta(1)$ queries to U and $U^\dagger$.

Without loss of generality, we will focus on learning the real component of the expectation value since H_j is Hermitian and unitary. The eigenvalues of the Grover oracle are then $\phi_j = \pm 2\sin^{-1}(\sqrt{[1 + \mathrm{Re}\langle\psi|H_j|\psi\rangle]/2})$. We can then learn these expectation values using phase estimation, which requires with high probability $O(1/\epsilon_j)$ applications of the Grover oracle to learn the eigenvalue to within error ϵ_j.

Now let us assume that we learn ϕ_j within error ϵ_j. It then follows that the inferred value of the expectation value satisfies

$$\widehat{\langle\psi|H_j|\psi\rangle} = 2\sin^2\left(\frac{\phi_j + \epsilon_j}{2}\right) - 1 \tag{32}$$

Since $\sin^2(\cdot)$ is a differentiable function, Taylor analysis shows that the error in $\langle\psi|H_j|\psi\rangle$ is

$$\left|\widehat{\langle\psi|H_j|\psi\rangle} - \langle\psi|H_j|\psi\rangle\right| \in O(\epsilon_j) \tag{33}$$

This shows that, up to a multiplicative constant, the error in the inferred expectation value is the error in the phase estimation step.

Now let us pick $\epsilon_j = \epsilon/|h_j|$. It then follows, from the triangle inequality, and the fact that the errors must be multiplied by h_j, that the total error in the calculation of the Hamiltonian expectation value is at most ϵ. The total number of applications of U and $U^\dagger$ required by the algorithm is then

$$O\left(\sum_j 1/\epsilon_j\right) \subseteq O\left(\sum_j |h_j|/\epsilon\right) \tag{34}$$

as claimed.

□

This scaling is significant because it shows that, in the worst case scenarios, the scaling of the algorithm is comparable (up to logarithmic factors) to IPEA being applied on top of the best known Hamiltonian simulation methods. Indeed, it can also be preferable because it does not require a full Trotter step to be applied. However, it does require much deeper circuits than traditional VQE, which limits its applicability in non-fault tolerant applications.

Therefore the ultimate reason for using IPEA in our algorithm is not because it provides better asymptotic scaling above VQE for estimating groundstate energies when amplitude estimation is employed. The best reason to use IPEA

over VQE is that it provides a projector onto the eigenvectors of the Hamiltonian, which is something that cannot be promised with a polynomial sized variational ansatz. This means that the final phase estimation in our algorithm should be viewed as a means of addressing shortcomings in the variational ansatz, and the VQE step should be seen as a systematic approach to addressing the state preparation problem in IPEA on a fault tolerant quantum computer.

section S4. Phase estimation without quantum collapse

A major drawback of quantum phase estimation is that, while it substantially reduces the depth of the entire inference process, it cannot be broken up into a series of short experiments. These long evolutions are needed in order to collapse a quantum state into a single eigenvector. Here we address this issue by introducing a new approach to phase estimation that does not require the quantum state to be collapsed; instead, we collapse the state classically.

The main idea behind our approach is to use a classical learning algorithm to patch together a series of experiments performed on identically prepared quantum states to learn a single eigenvalue. If we have a single eigenstate fed into the IPEA circuit then the probability of measuring "0" is

$$P(0|\lambda_j; t, \phi) = \cos^2((\lambda_j - \phi)t/2) \tag{35}$$

since this is a Bernoulli experiment $P(1|\lambda_j; t, \phi) = 1 - P(0|\lambda_j; t, \phi)$. These probabilities are also referred to as the likelihood function Bayesian approaches to phase estimation use the likelihood function to update a probability distribution, known as a "prior" distribution, that describes the current state of knowledge of the eigenphase. Approximate methods such as the rejection filter phase estimation *(40)* or sequential Monte-Carlo algorithms have been successfully used to solve this problem.

However, if we have an initial state of the form $|\psi\rangle = \sum_{j=1}^{2^n} \alpha_j |\lambda_j\rangle$ where $|\lambda_j\rangle$ are eigenstates of U then the likelihood function takes the form

$$P(0|\lambda_1, \ldots, \lambda_{2^n}; t, \phi) = \sum_{j=1}^{2^n} |\alpha_j|^2 \cos^2((\lambda_j - \phi)t/2) \tag{36}$$

If we want to apply standard approaches to learning the groundstate energy, λ_1, then we need to store all 2^n eigenvalues in order to perform the update properly. This is of course intractable for large n and a natural approach to combat this is to truncate the space.

Our approach here is to truncate the space by only inferring a single eigenvalue from such experiments by marginalizing over the λ_j that do not correspond to the ground state. This yields a likelihood function of the form

$$P(0|\lambda_1; t, \phi) := 1/(2\pi)^{2^n - 1} \int P(0|\lambda_1, \ldots, \lambda_{2^n}) \mathrm{d}\lambda_2 \cdots \mathrm{d}\lambda_{2^n} = |\alpha|^2 \cos^2((\lambda_1 - \phi)t/2) + (1 - |\alpha|^2)/2 \tag{37}$$

where the parameter α is key for this algorithm working properly. Bayesian inference can be adopted to learn λ_1 using adaptively chosen experiments, such as with the heuristic proposed in *(40)*.

We then solve the inference problem by using a modified version of rejection filter phase estimation (RFPE). The algorithm works by discretizing the prior distribution over P points and then performs approximate Bayesian inference on the discrete distribution. The posterior mean and variance are computed and then the posterior is replaced by a Gaussian distribution with the same mean and standard deviation. The interval used is then rescaled and shifted to accommodate the new distribution. This process is repeated until the posterior variance becomes sufficiently small.

This process has one key advantage over exact Bayesian inference. The ability for the algorithm to exclude hypotheses allows it to remain stable even if the support of the initial state is broad in the eigenbasis of U. In order to see this, consider an experiment with two eigenvalues and assume that the prior distribution is a sharply peaked Gaussian about λ_1 with $\sigma \ll |\lambda_1 - \lambda_2|$. If this is the case and a datum D is observed that corresponds to λ_2 then the likelihood function corresponding to this hypothesis is $\cos^2((\lambda_2 - \phi)t/2)$. Since $t = 1/\sigma$, $|\lambda_2 - \phi|t \gg 1$ and in general as t increases will be uniformly distributed, mod 2π, over $[0, 2\pi)$. This means that the shift in the posterior arisen from this hypothesis will be random and because the prior has no support over the hypothesis, such updates will not validate λ_2. Instead these updates randomly perturb the posterior distribution for λ_1, which given α is chosen appropriately small will not have a net effect on the learning. On the other hand, if the datum was taken from λ_1 then this argument no longer holds because $|\lambda_1 - \phi|/\sigma$ will be of order unity with high probability. Thus we expect such an inference algorithm to be capable of learning despite the presence of additional eigenvalues.

We see in Fig. 1 that this in fact does occur. Two eigenvalues are considered and the initial state is chosen such that there is a uniform distribution over each eigenvalue. We choose a uniform distribution because it is maximally pessimistic. We then repeat the inference algorithm for a fixed number of iterations and compute the minimum difference between the posterior mean and either of the eigenvalues. We see that despite the fact that half the data comes from a different eigenvalue, the algorithm is able to rapidly learn one of the eigenvalues exponentially quickly. The eigenvalue

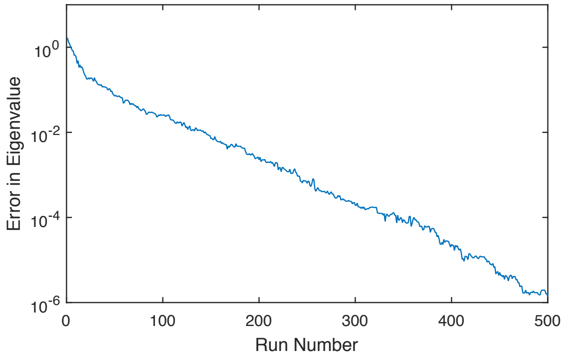

fig. S1. **Median inference error in eigenvalue inference with $\alpha = 1/2$ for a distribution with two randomly chosen eigenvalues and likelihood function (37) is used.**

it outputs is random with probability 1/2 of outputting either eigenvalue. This verifies that quantum wave function collapse is not needed to infer eigenvalues and that the data can be classically "collapsed" by introducing biases into the approximate Bayesian inference algorithm.

section S5. Experimental details

S 5.1 The Silicon quantum photonic device

The silicon-on-insulator (SOI) quantum photonic chip was fabricated on a silicon-on-insulator material system using 248 nm deep-UV photolithography and dry etching a mass manufacturable approach. Single-mode waveguides were designed with a width of 450 nm and thickness of 220 nm, and covered with a $1\mu m$ silicon dioxide upper cladding. Spot-size converters with 300 μm long inverse taper with a 200 nm wide tip beneath a 4×4 μm^2 polyimide waveguide were used to couple photons into and out of the device. Approximately, a 8 dB coupling loss per facet was observed between the waveguide and single-mode lensed fiber with a 3 μm mode-diameter. Passive beam-splitters with near 50% reflectivity were realized using multi-mode interferometers (MMIs) couplers with a footprint of 2.8 $\mu m \times 28$ μm. The waveguide crossing in the device showed a -40 dB crosstalk between two waveguides, substantially negligible for the purpose of this work.

This device integrates key functionalities for photonic quantum information processing, such as photonic qubit quantum gates and photon-pair sources. The two spirally-wrapped waveguide sources with a 1.2 cm length were designed to generate single photon-pairs by spontaneous four-wave mixing (SFWM) *(27, 53)*. The photonic qubits in this device are realized through path encoding, as shown in *(28, 29, 31)*. Such encoding allows easier reconfigurability, compared for example with polarization encoded qubits *(30)*. The structure of the entangled source is equivalent to that one presented in *(31)*, and is used to generate and entangled, path-encoded two qubit states, see SM 4.

The reconfigurability relies on thermo-optical phase shifters, which consists of metal resistive heaters on top of the silicon waveguides isolated by a dioxide layer in between. The heat locally dissipated in the waveguide induces the refractive index change responsible of the phase-shift. The heaters can be independently driven and controlled by home-designed multi-channel electronic. Each heater has independent connections for voltage supply but not for the ground that instead was common to all the heaters, causing electrical cross-talk between them. In order to drastically reduce the electrical cross-talk the phase shifters were driven in current. The heater driver board was composed of 12 bits DAC connected to amplifiers and driven through RS-232 interface. This allows to control the phase with a precision of $\simeq 0.01$ rad in our experiment. Thermal cross-talk was observed between the different degrees of freedom of the silicon quantum photonic device, the effect of which was partially compensated by performing different calibrations for different chip configurations.

S 5.2 Experimental Setup

A schematic description of the experimental set-up is reported in fig. S2. The silicon quantum photonic chip was mounted and wire-bonded on an electronic PCB that was glued by thermal epoxy on a Peltier device connected to a heat-sink and controlled by a PID algorithm to stabilize the temperature of the chip. The input/output optical fibers were automatically recoupled by maximizing the coupling efficiency between waveguides and fibers.

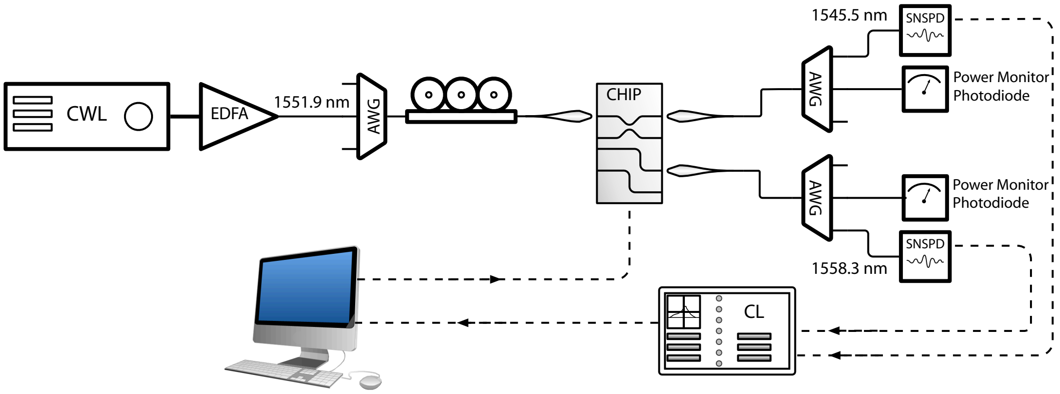

fig. S2. Schematic representation of the experimental setup. The light from a CW laser light source is amplified through an EDFA. The pump is filtered through an AWG and the polarization adjusted through a manual polarization controller, then focused inside the chip and collected by lensed-fibres. The emerging light is then filtered through AWG, where the single photons channel and the pump are separated. While the CW light is detected by standard photo-diodes for monitoring the coupling, the single photons are detected through super-conductive nano-wire single photon detectors SNSPDs, whose pulses are analyzed by a time interval analyzer connected to a classical computer. The classical computer is also used to control the on-chip thermo-optic phase shifters.

A continuous-wave (CW) bright laser at 1551.9 nm, amplified by an erbium doped fiber amplifier (EDFA), with an emerging power of $\sim$ 10 mW, was used to pump the two sources for producing photon pairs, i.e. *signal* and *idler* photons, by spontaneous four-wave mixing (SFWM) *(27)*. The use of a fiber-polarization controller before the silicon device ensured that the transverse-electric (TE) polarized CW light was injected into the device, after the background was removed through the use of array waveguide gratings (AWG) wavelength division multiplexers (WDM). The output SFWM-generated photons in the quantum device were separated apart from the pump light by off-chip AWG with a 0.9 nm bandwidth and $>$ 90 dB extinction. We chose the signal and idler photons at the wavelength of 1545.5 nm and 1558.3 nm respectively. Photons were routed into single-mode fibers and finally detected using superconducting nano-wires single-photon detectors (SNSPDs) from PhotonSpot^{TM}, with an average efficiency of 85%, 100Hz dark counts and 50 ns dead-time. The coincidence counts were recorded by the use of a time interval analyzer (Picoharp 300 by PicoQuant^{TM}) with an integration time of 10 s per data point and a coincidence window of approximately 400 ps. A maximal photon-pair rate of about 150 Hz was observed.

The CW light and single photons were used to characterize the single-qubit gates composing the state preparation, unitary operation, and quantum state tomography. Using the CW light, we measured the mapping between the injected current and electronic power consumption, and between the electronic power and optical power, from which we linked the current and phase for each phase-shifter. A constrained least squares algorithm was used to fit the experimental data. The characterization of each part of the device was performed for different configurations of the remaining parameters, in order to properly compensate for thermal cross-talk. Both λ-classical interference and $\lambda/2$-quantum interference, shown in Fig.2 inset, confirm the high quality of the device and its characterization. Interference plots were obtained for the control qubit by setting the final MZI in the corresponding register as a Hardamard gate (i.e relative phase shift of $\pi/2$) and collecting photon coincidences in the top two modes of the device (same procedure can be performed for the target qubit).

S 5.3 Controlled unitary operations

The implementation of WAVES requires a quantum device able to implement arbitrary controlled unitary ($C\hat{U}$) operations. The difficulty to implement arbitrary $C\hat{U}$ gates in quantum photonics has limited first implementations of quantum algorithms to particular classes of problems with specific symmetries *(11)*. In this experiment we were able to implement an arbitrary $C\hat{U}$ using an entanglement-based scheme which does not require pre-decomposition *(32)*. We report for the first time its adoption in integrated photonics. In this section we describe the implementation of this scheme.

Following figure 2 in the paper, the state generated by spontaneous four wave mixing (SFWM) in the two spiral sources, considering only 2 photon generation events, is given by $(|0200\rangle + |0020\rangle)/\sqrt{2}$. After the MMIs splitters and the central crossing the full state becomes $(|2000\rangle + |0200\rangle + |0020\rangle + |0002\rangle + 2|1010\rangle + 2|0101\rangle)/\sqrt{8}$. Using the

post-selection scheme *(31)*, only the part corresponding to a Bell state is picked up and used for the computation.

$$\frac{|1010\rangle + |0101\rangle}{\sqrt{2}} \tag{38}$$

The disregarded part of the state $(|2000\rangle + |0200\rangle)/\sqrt{2}$, which can be filtered out by post-selection of the output photons in the control qubit modes, was used to test the quality of the sources and circuits. By setting the tomography stage of the control qubit as the Hadamard rotation with a continue rotation around the Pauli z basis $\hat{R}_z(\theta)$, we observed quantum interference with a visibility of 1.00 ± 0.02 (see inset in Fig.2 of the main text).

Labeling the logic state $|0\rangle$ ($|1\rangle$) for the top (bottom) path of each photon, referring to Fig.2 of the main text, the input Fock state can be rewritten as the qubit state $(|0\rangle_C|0\rangle_P + |1\rangle_C|1\rangle_P)/\sqrt{2}$. Two additional modes are added in the bottom paths, which can be represented as the addition of another qubit, which is our target qubit, giving the state $(|0\rangle_C|0\rangle_T|0\rangle_P + |1\rangle_C|1\rangle_T|1\rangle_P)/\sqrt{2}$. For both bottom paths the two modes are prepared in the same input state $|\psi\rangle_T$, using the operations reported in the main text. The state is later evolved according to $\hat{I}$ in the upper path and $\hat{U}$ in the lower one, giving the total state

$$\frac{|0\rangle_C|\psi\rangle_T|0\rangle_P + |1\rangle_C(\hat{U}|\psi\rangle_T)|1\rangle_P}{\sqrt{2}} \tag{39}$$

At this point, using a waveguide crossing and two integrated beam-splitters we erase the path information between the upper and the lower path, which is equivalent to apply an Hadamard gate to the third qubit, obtaining

$$\frac{(|0\rangle_C|\psi\rangle_T + |1\rangle_C\hat{U}|\psi\rangle_T)|0\rangle_P + (|0\rangle_C|\psi\rangle_T - |1\rangle_C\hat{U}|\psi\rangle_T)|1\rangle_P}{2} \tag{40}$$

Post-selecting the cases where the target photon emerges from one of the top modes, i.e. projecting in $|0\rangle_P$, we finally get the output state

$$\frac{|0\rangle_C|\psi\rangle_T + |1\rangle_C\hat{U}|\psi\rangle_T}{\sqrt{2}} \tag{41}$$

which gives the desired arbitrary control-unitary operation.

We remark that applying other operations after the $\hat{U}$ this scheme also allows to cascade different control-unitary operations in a non-compiled way. This can be used, for example, to perform Trotterization for the time evolution. However the probabilistic nature of this scheme (due to the post-selection) makes it not scalable.

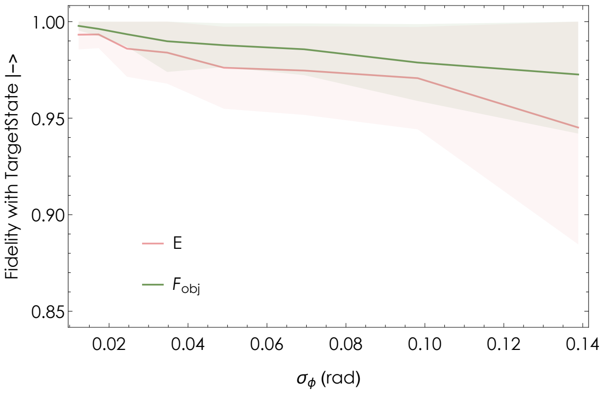

fig. S3. Numerical simulations of the variational ground state search robustness for the bosonic one-qubit $\hat{H}$ against gate infidelities using $\mathcal{F}_{\mathbf{obj}}$ or $\mathcal{E}$ alone. Infidelities in the gate implementation is modeled as Gaussian noise in the phase shifting components of the photonic circuit. Average fidelities with the true eigenstate $F_{|-\rangle}$ (solid lines) along with 67.5 % confidence intervals (shaded areas) are reported after algorithm's convergence.

section S6. Robustness against experimental noise of the variational search

In this section we investigate numerically the effect of noise in the WAVES variational search. This paragraph focuses upon the ground state search case experimentally performed in this paper, where noise affects both estimators $\mathcal{E}$ and $\mathcal{P}$, guiding the variational search of the Hilbert space. We introduce two different sources of noise, expected to play a major role in our setup:

table S1. Summary of ansätze used for simulations in the main paper and in the Supplementary Materials for the various systems investigated, along with the cardinality of their parameterization, dim $(\vec{\theta})$. PH refers to the parametrized Hamiltonian ansatz in Methods Eq. (7). We report in this same table also the number of excitation operators - $\dim(\{\hat{E}_{p_i}\})$ - that have been tested in numerical simulations and the reference state for the system $|\Phi\rangle$.

	truncated PH $\dim(\vec{\theta})$	standard PH $\dim(\vec{\theta})$	unrestricted UCCSD $\dim(\vec{\theta})$	$\dim(\{\hat{E}_{p_i}\})$	$\|\Phi\rangle$
H_2 (4 qubit)	13	15	110	3	$\|1100\rangle$
H_3^+ (6 qubit)	60	66	462	15	$\|111000\rangle$
H_3 (6 qubit)	112	114	N.A.	15	$\|110000\rangle$
H_4 (8 qubit)	181	185	N.A.	22	$\|11110000\rangle$

- Poissonian noise arising from coincidence events counts (see also SM S 2.1).
- Infidelity in the gates implementation (i.e. uncertainty in the phases implemented by the thermo-optical phase shifters on chip), affecting both the state preparation and the $C\hat{U}$ implementation.

Studying a noise model for this implementation of WAVES also allows to address whether the proposed objective function $\mathcal{F}_{\text{obj}}$ is capable of providing any advantage (or disadvantage) in the robustness of the search, compared to the adoption of a variational search based upon the energy estimator alone (therefore, equivalent to previous VQE ground state search demonstrations *(10, 11, 12)*). Data from 100 averaged numerical simulations, running the variational search with either $\mathcal{F}_{\text{obj}}$ or a simplified objective function $\mathcal{F}'_{\text{obj}} = \mathcal{E}$, are reported in fig. S3.
When Poissonian noise alone is considered discrepancies observed between the performances of $\mathcal{F}_{\text{obj}}$ and $\mathcal{F}'_{\text{obj}}$ are small, and any difference among the two approaches falls within the (statistical) error bars. For this kind of noise, therefore, nothing can be concluded in favor or against the two approaches.

Infidelity in the implementation of gates, for a digital quantum simulator without error correction, is a particularly critical feature *(54)*. In order to simulate noise affecting the qubit operations, for each thermo-optical phase shifter manipulating the target qubit we replaced the correct phase $\bar{\varphi}_i$ – required to implement the transformation – with a synthetic value φ, sampled from a Gaussian distribution $\varphi_i \sim \mathcal{N}(\bar{\varphi}_i, \sigma_\varphi)$. As σ_φ increases, the behavior of the simulated physical circuit deviates from the ideal case. This is a realistic way to simulate such noise in our photonic setup. Each phase shift implemented is proportional to the power applied to heating element, in turn affected by electrical and thermal fluctuations. A thorough characterization is instead expected to avoid systematic biases in some of the phases $\{\bar{\varphi}\}$. Phases involved in the control register have been excluded from this simulation. Indeed, the scope was to emphasize the role of imperfect qubit operations in the target manipulation, while neglecting contributions from errors in the tomography of the control qubit.
This noise model applied to experimental conditions led to the results reported in Fig. 3 C-F of the main text, reproducing quite accurately the experimental findings. In that case, according to device characterization data, it is assumed $\sigma_\varphi \simeq 0.012$ *rad* in the simulations. Slight differences between the predictions and the experimental data can be likely explained via uncharacterized residual thermal cross-talk between the phase shifters (not taken into account in the model), that contributes to systematic errors in the implemented phases. A further investigation of how disruptive such gate infidelities may be in noisier implementations is instead provided in fig. S3 for the bosonic Hamiltonian experimentally investigated in the main text. For this kind of noise, adopting the objective function $\mathcal{F}_{\text{obj}}$ appears a consistently more robust strategy than using $\mathcal{E}$ alone as an estimator: a difference that increases with the noise level. In particular, already for $\sigma_\varphi \simeq 0.14$ the final fidelity achieved in the search with $\mathcal{E}$ alone falls below $F_{|-\rangle} = 95\%$, whereas $\mathcal{F}_{\text{obj}}$ keeps providing accurate ground state estimates.

section S7. Numerical simulations of hydrogen molecules

We expand in this paragraph the numerical simulations beyond the case experimentally tested, in order to investigate the capability of WAVES to detect different eigenstates for systems of dimensionality as high as 8 qubits. Simulations of the WAVES algorithm are here performed at a logical circuit level, unlike the simulations in SM S 2.1 and S 6, where a model for the photonic circuit behavior had been adopted for the simulations. However, all the results presented in this section include a binomial noise model for the control qubit tomography (conceptually equivalent to the Poissonian noise model for a photonic setup). The latter one was introduced in SM S 6. The level of noise chosen in the simulations is compatible with what obtained in a standard experimental run for the 1 qubit case (i.e. 1500 measurements for each control qubit tomography).

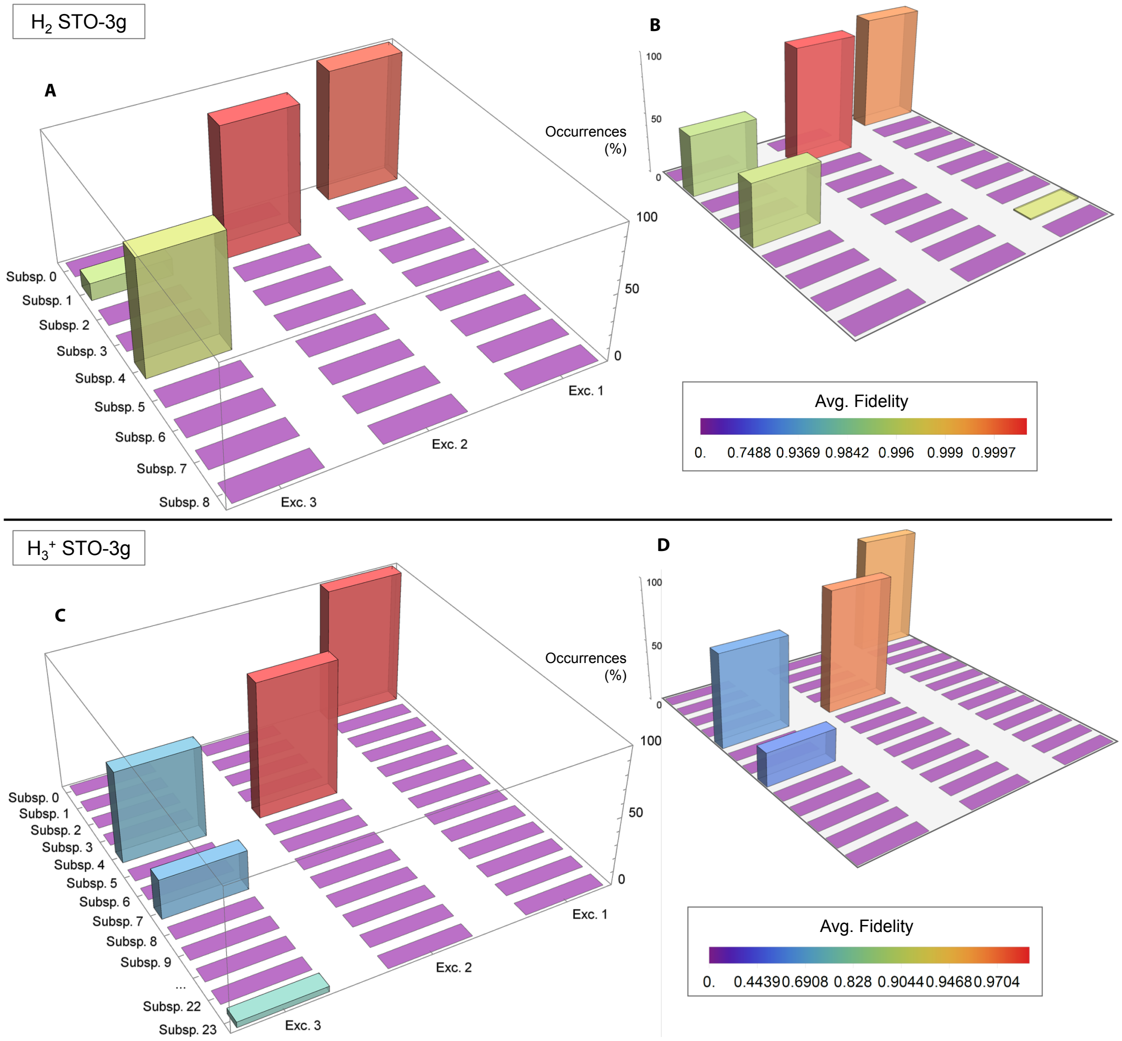

fig. S4. Synopsis of numerical simulations of excited states searches for the molecules H_2 and H_3^+, adopting the full PH ansatz (**A&C**). Results for the synthetically reduced ansatz are in the inset outlined in light gray (**B&D**). On one horizontal axis are reported the non-degenerate subspaces spanned by the eigenstates of the corresponding Hamiltonian ('*Subsp. i*'), which are 9 for H_2 and 23 for H_3^+. On the other axis are the 3 different excitation operators of the form E_p ('*Exc. j*') tested. Height of the histogram with coordinates $\{i, j\}$ corresponds to the relative frequency with which the swarm of particles collapses onto the i-th excited subspace, when $\hat{E}_{p_j}$ is adopted in the second phase of the search. We accept the swarm has collapsed onto the $\mathbb{E}_i$, whenever the corresponding projector $\prod_i$ exhibits the highest overlap with the estimate $|\Psi\rangle$ at the end of the search (see Supp. Eq. 42). Fidelities reported for a certain subspace $\mathbb{E}_i$ and $\hat{E}_{p_j}$ are calculated averaging the final fidelity achieved by all and only those algorithm runs, where the swarm had collapsed onto $\mathbb{E}_i$. The bottom-right bar legend explicates the color coding.

In all cases we adopt the protocol as described in Fig. 1 of the main text. That is, we first variationally optimize the parametrization $\vec{\theta}$ provided by one of the ansätze in table S1 learning the optimal $\vec{\theta}_g$ to prepare the ground state. For this search, we make no use of a-priori knowledge about the ground state: a reference state for the system is the initial guess for the ground state, i.e. $\hat{A}(\vec{\theta}_0)|\Phi\rangle \equiv |\Phi\rangle$ in Eq. (9) of Methods. We draw the initial swarm of particles from a Gaussian distribution centered around 0 for each parameter θ_i, with a standard deviation chosen arbitrarily to be much bigger than $\max\{|\theta_i|\}$. For the different molecular systems analyzed, this is equivalent to having very different average initial overlaps with the ideal ground state $|\Psi_g\rangle$, as calculated diagonalizing the corresponding Hamiltonian. Precisely, $|\langle\Psi_0|\Psi_g\rangle|^2$ is in the range $0.7 - 0.8$ for H_2 and H_3^+, but as low as 0.2 for both H_3 and H_4. Even when starting from such poor initial guess states, WAVES was capable of achieving fidelities higher than 95% with the ideal target state . In those cases where a better initial overlap bootstrapped the variational search, final fidelities observed where in excess of 99%.
This discrepancy in the fidelities shall not be misinterpreted as a fundamental limit deriving from the quality of the initial guess provided to WAVES. Indeed, it is worth mentioning that in order to simulate realistic experimental conditions for near-term WAVES implementations, not only we included a noise model in the control-qubit tomography, but we restricted the number of particles in the swarm as equivalently done in SM S 2.1. This kept execution times for the protocol within reasonable limits for state-of-art photonic implementations. E.g. the longest 8 qubit simulations converged after performing only about 500 single-qubit tomographies when adopting 20 particles in the swarm optimization performed over more than 400 parameters. In comparison with $\sim$100 tomographies required by our experimental proof of concept (where 8 particles in the swarm optimized over 2 parameters).

table S2. Summary of possible situations occurring in numerical simulations when WAVES is performed with different ansätze and excitation operators, **targeting a certain excited subspace $\mathbb{E}_\tau$ from an initial guess** $|\Psi_0\rangle$.

	Ansatz allows $|\Psi_0\rangle \to \mathbb{E}_{i(\tau)}$	Ansatz prevents $|\Psi_0\rangle \to \mathbb{E}_{i(\tau)}$
$|\Psi_0\rangle$ provides accurate guess for $\mathbb{E}_\tau$<br>$\langle\Psi_0|\prod_i|\Psi_0\rangle \ll \langle\Psi_0|\prod_\tau|\Psi_0\rangle, \forall i \neq \tau$	✓<br>(convergence to $\mathbb{E}_\tau$)	N.A.
$|\Psi_0\rangle$ provides no accurate guess $\mathbb{E}_\tau$<br>$\exists\, i \neq \tau : \langle\Psi_0|\prod_i|\Psi_0\rangle \simeq \langle\Psi_0|\prod_\tau|\Psi_0\rangle$	✓<br>(convergence to either $\mathbb{E}_\tau$, $\mathbb{E}_i$)	✗<br>(no convergence to any $\mathbb{E}_i$)

A wide variety of behaviors can be observed when performing WAVES for excited state searches. We remind that in our protocol this is preferentially achieved by applying a set of different excitation operators of the form $\hat{E}_p$ to the ground state parametrization $\hat{A}(\vec{\theta}_g)|\Phi\rangle$ obtained in the first step. An additional variational search is then performed starting from this initial guess $|\Psi\rangle$, adopting $T \gg 1$ in $\mathcal{F}_{\text{obj}}$ (see Methods "Excitation operators for Chemical Hamiltonians"). Specifically for this set of simulations where a swarm optimization is performed, the initial set of particles was initially drawn from a Gaussian distribution centered around θ_{i_g} for each parameter θ_i, with an initial standard deviation approximately equal to $\max\{|\theta_i|\}$. When targeting a subspace of degenerate excited states, we replace the fidelity $|\langle\Psi_0|\Psi_\tau\rangle|^2$ for the generic trial state $|\Psi\rangle$ with a generalized expression, given by the projection onto $\mathbb{E}_\tau$

$$F = \Big\langle\Psi\Big|\prod_\tau\Big|\Psi\Big\rangle = \Big\langle\Psi\Big|\sum_k(|\Psi_{\tau_k}\rangle\langle\Psi_{\tau_k}|)\Big|\Psi\Big\rangle \tag{42}$$

where $\{|\Psi_{\tau_k}\rangle\}$ is a basis of $\mathbb{E}_\tau$. We also assume for the scope of this work that IPEA up to 32 bits of accuracy will refine the eigenvalue estimate provided by the first step of the WAVES protocol. Consequently, targeting spectroscopical accuracy, energy differences smaller than 10^{-9} *Ha* will be ignored and corresponding eigenstates will be considered degenerate. Finally, in order to simplify the interpretation of the data for each variational step, we also simplistically adopt $\mathcal{F}_{\text{obj}} \equiv -\mathcal{P} \equiv \mathcal{S} - 1$. In fig. S4A we report a summary of the results for excited state searches in the H_2 system, using 3 different excitation operators $\hat{E}_{p_j}$, and measuring projectors onto all the 9 non-degenerate excited subspaces that characterize the corresponding Hamiltonian. The set of $\hat{E}_{p_j}$ is expected to provide access to a portion of the spectrum with most of its support lying onto low–energy excited states, and a limited support onto higher energy excited states. We notice how the optimization leads to a final fidelity F with one of the excited subspace(s) exceeding 99%+ in all cases (color-coded). In particular, E_{p_3} produces an initial guess that is a superposition of eigenstates belonging to the 1st and 4th excited subspaces. This is a highly non-trivial case: because of the initial standard deviation adopted in the algorithm, the initial swarm of particles spans fidelities that are as low as 0.5 with either subspace. Performing IPEA directly with this initial guess would perform poorly. As emphasized in SM S 7.2 adopting a Folded Spectrum method would also be of little help in this case, if insufficient information about the energy spectrum is available.
WAVES instead refines the initial guess detecting 87% of the times the 4th subspace. This preferential collapse approximately matches what expected from the initial guess $|\Psi_0\rangle$, that has 0.78 overlap with the 4th subspace.

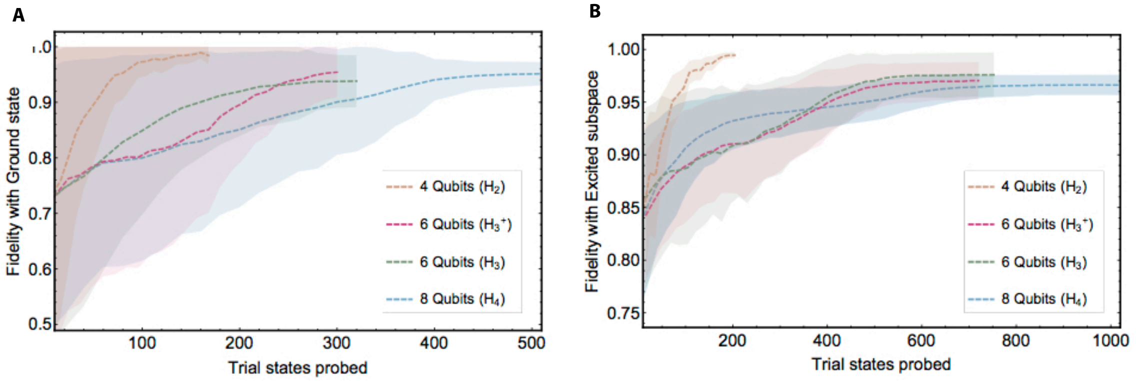

fig. S5. Numerical simulations of the WAVES variational search for the synthetically truncated PH ansatz is studied in the molecular hydrogen systems (H_2, H_3^+, H_3, H_4). (**A**) Variational search for the ground state. (**B**) Variational search for the targeted subspace of degenerate excited states with initial excitation perturbation $\hat{E}_{p_i}$. The average fidelities achieved by the particle swarm optimization are calculated for 100 independent runs of WAVES. Dashed lines are average fidelities. Shaded areas indicate 67.5% confidence intervals. On the x-axis we refer to the cumulative number of trial states probed, i.e. the number of particles in the swarm times the variational steps. For ease of comparison, the x-axis origin has been shifted in (**A**) for the various cases in order to have equivalent fidelity for the average initial guess. In all simulations a binomial noise model has been taken into account when performing projective measurements.

Equivalently, in fig. S4C are summarized the results for the H_3^+ molecule. The bigger dimensionality of this system leads to 23 non-degenerate excited subspaces. Among the cases investigated, we report only 3 different excitation operators, with the intent to reproduce a situation similar to what observed for H_2, and are thus relabeled as *'Exc. 1-3'*. The optimization leads to a final fidelity F with one of the excited subspace(s) exceeding 99%+, in each case where the initial guess accurately targets a single excited subspace. For excitation operator E_{p_3}, the preferential collapse of the particle swarm into either of the subspaces 4, 7 and 23 follows well what expected from the initial overlaps of $|\Psi_0\rangle$. We remark how a successful variational search for this last case required the adoption of a more accurate ansatz (the UCCSD in table S1). E_{p_3} produces an initial guess that is a superposition of eigenstates belonging to 7 different excited subspaces, yet predominantly targeting the 4th excited-subspace (that has the highest overlap with the initial guess $|\Psi_0\rangle$). In order to access this whole portion of the Hilbert space and achieve a successful convergence, the simple PH ansatz would not suffice - see fig. S7. As shown in SM S 1.1, it is certainly possible for WAVES to provide a sanity check for the final eigenstate estimate obtained. In the same figure we outline how this may be performed in this case, making use of the eigenstate witness $\mathcal{S}$ and increasing the evolution time t (for simplicity, we assume to operate always within a range where the Trotterization error is expected to be negligible). If no hardware decoherence issues arise for such longer evolution time, consistent degradation in the final $\mathcal{S}$ obtained at the end of a single run indicate a failure: either the ansatz has to be enriched, either the initial guess perturbed, for WAVES to achieve a successful convergence to a single $|\Psi_\tau\rangle$ or $\mathbb{E}_\tau$.
We will not report for brevity further analyses for the H_3 and H_4 systems, being the behavior of WAVES in this case equivalent to findings already discussed for smaller systems.

S 7.1 Synthetically reduced ansätze

One of the arguments often raised against variational methods is that they strongly rely on the accuracy of the ansatz chosen for the variational state preparation.

When considering more correlated and complex systems the accuracy of such ansatz is likely to be heavily limited. For this reason, trying to characterize variational methods in the presence of inaccurate or incomplete ansätze is of fundamental importance for the assessment of their realistic performances.

In the previous SM section we presented and discussed simulations mostly obtained with a standard PH ansatz, expected to achieve a reasonable accuracy in describing weakly correlated systems. Here we compare such results with a truncated ansatz $\hat{A}'$, obtained after a gradual removal of those terms in the PH ansatz, corresponding to the smallest values $|\theta_g|$. A more detailed breakdown of the procedure is reported in Algorithm 2.

Algorithm 2: Protocol for the synthetic truncation of the ansatz

Data: Parametrized ansatz $\hat{A}(\vec{\theta}) \equiv \sum_{i\in I}(\theta_i \hat{A}_i)$, excitation operator $\hat{E}_{p_\tau}$ and targeted excited eigenstate $|\Psi_\tau\rangle$, threshold $\tilde{F}_0$ for the initial guess

obtain from WAVES optimal parameters for the ground state $\hat{A}_g = \hat{A}(\vec{\theta}_g)$, minimizing $\mathcal{F}_{\text{obj}} = -a\mathcal{P} + b\mathcal{E}$;

calculate $|\Psi_0\rangle = \hat{E}_{p_\tau}\hat{A}_g|\Phi\rangle$;

initialize $I' = I$;

while $|\langle\Psi_0|\Psi_\tau\rangle|^2 \geq \tilde{F}_0$ **do**

  find ι: $\theta_{g_\iota} = \min_i(\{|\theta_{g_i}|\})$;

  $I' \leftarrow I' - \iota$;

  $\hat{A}'_g = \sum_{i\in I}(\theta_{g_i}\hat{A}_i)$;

  calculate $|\Psi_0\rangle = \hat{E}_{p_\tau}\hat{A}'_g|\Phi\rangle$;

end

Result: truncated ansatz $\hat{A}'(\vec{\theta}) \equiv \sum_{i\in I}(\theta_i\hat{A}_i)$, with $\dim(I') < \dim(I)$

$\hat{A}'$ thus simulates those cases where only limited knowledge on the physical system is available. The results are summarized for both ground and excited state searches in fig. S5 A&B, respectively. To stress the resilience of WAVES to a poor initial guess for the parameters, all the simulations in fig. S5 were performed assuming no *a-priori* knowledge of optimal $\vec{\theta}$, i.e. the initial guess $\hat{A}(\vec{\theta}_0)|\Phi\rangle \equiv |\Phi\rangle$. For all the cases investigated, the variational search in WAVES is able to consistently identify both the ground and targeted excited states. Fidelities of approximately 95% are achieved, even for cases where the initial guess retained only small overlap with the targeted excited state and the incomplete ansatz reduces the accuracy in the stat preparation.

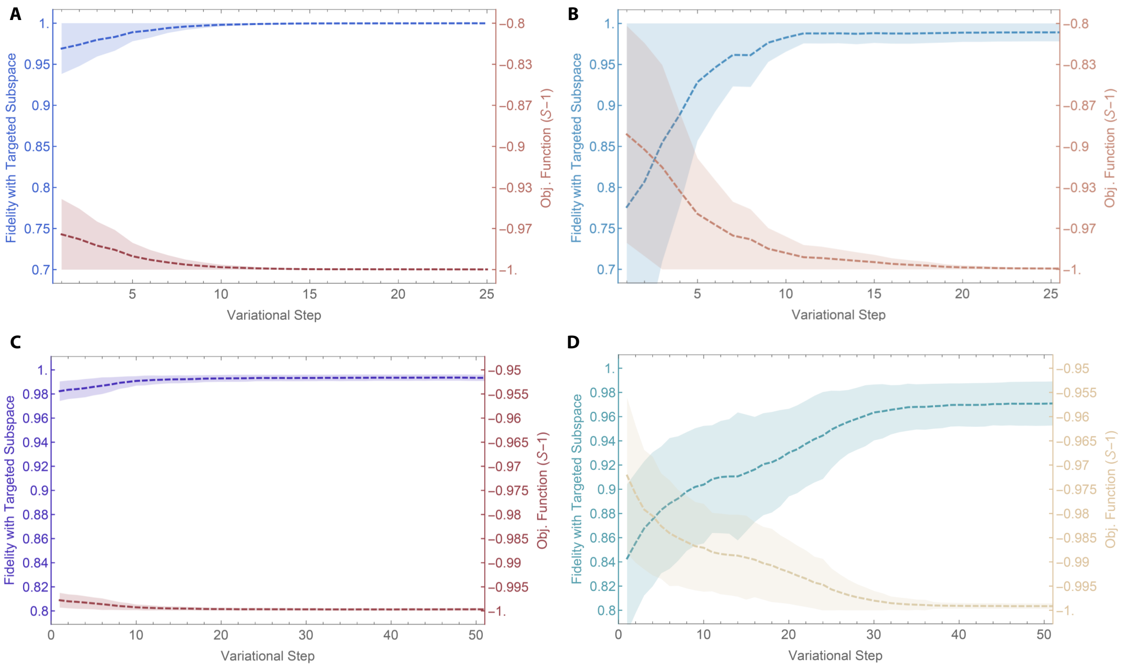

fig. S6. Convergence of the WAVES algorithm to a subspace of excited states in the case of the H_2 STO-3g (**A**-**B**, upper row) and H_3^+ STO-3g (**C**-**D**, lower row). On the left (A&C) we report simulations averaged over 100 runs of the algorithm, adopting the standard PH ansatz. On the right (B&D, emphasized by fainter colors), the algorithm is run with identical parameters, but adopting a truncated version of the same ansatz (refer to Algorithm 2). Respectively 2 and 6 operators were truncated in cases B&D.

Details of the variational search for excited states in the H_2 and H_3^+ molecules are reported in fig. S6 A&C (B&D) respectively, comparing simulations with the standard (truncated) version of the PH ansatz (see also table S1). A decrease in the fidelity and uncertainty of the final estimate upon convergence are observed in the truncated versus the untruncated ansatz (compare also the color-coded fidelities in fig. S4). Also, the restricted ansatz starts from a poorer

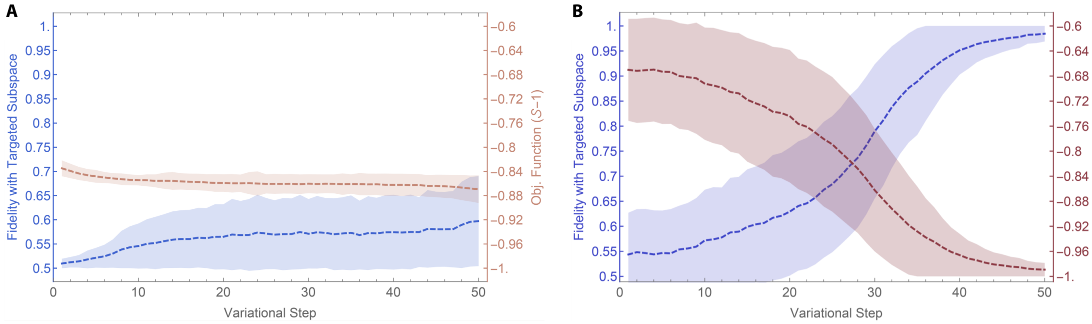

fig. S7. In **A**, we report the average results from 100 runs of the first part of the WAVES protocol adopting $\hat{E}_{p_3}$ for the PH ansatz in the H_3^+ system and $t = 10$ in Methods Eq. (9). The average entropy for the final estimate is $\mathcal{S} > 0.12$. In **B** an equivalent optimization adopts the unrestricted UCCSD ansatz from table S1, but the same swarm-optimization parameters as in Algorithm 1. A difference can be noticed in the 67.5% confidence intervals for both F and $\mathcal{S}$ at Step 0. This is due to attributing the same initial uncertainty to each variational parameter θ_i, being $\dim \vec{\theta}$ in the unrestricted UCCSD ansatz about 8 times larger than in the standard PH case.

initial guess and requires more variational steps to converge to the optimal value. Yet the algorithm displays a good resilience against such slightly inaccurate ansätze, being still capable to detect the excited subspaces with high fidelity. Again for the H_2 system, in the inset of fig. S4B, we observe how the reduced accuracy of this synthetic ansatz and overlap with the targeted eigenstate diminish the tendency of WAVES to collapse into the closest excited subspace.

For the more complex case of the H_3^+ system, the difference in the final fidelities achieved, as well as in the frequency with which the swarm collapses into either one of the excited subspaces $\mathbb{E}_i$ are also summarized in fig. S4D.

A specific case is displayed in fig. S6 C&D (corresponding to '*Exc. 2*'). An accurate initial estimate $|\Psi_0\rangle$ leads to a very rapid convergence to fidelities with $\mathbb{E}_4$ exceeding 99% for the PH ansatz in (C). In case (D), some of the crucial operators to explore the Hilbert space in proximity of the targeted subspace of excited have been evidently removed. This leads to a suddenly impoverished $|\Psi_0\rangle$, a consequently slower convergence, as well as a suboptimal fidelity with the targeted subspace $F \simeq 96\%$.

S 7.2 Comparison with folded spectrum method

We implemented a folded spectrum(FS)-like variational search *(55)*, where we search for the state $|\Psi\rangle$ minimizing the energy *folded* around the energy shift ϵ, as in the modified eigenvalue Supp. Eq. (43)

$$(\hat{\mathcal{H}} - \epsilon)^2|\Psi\rangle = (\mathcal{E} - \epsilon)^2|\Psi\rangle \tag{43}$$

which leads to the eigenstate closest in energy to the chosen ϵ. Here we replace the usual conjugate gradient optimization method adopted in FS *(56)* with a variational search for the state adopting the same particle swarm method in Algorithm 1, so to have a fair comparison ground with equivalent simulated runs of WAVES.
In this paragraph we report the results of applying FS to find excited states when applying the excitation operator $\hat{E}_{p_3}$ to the reference state of the H_2 systems, adopting a PH ansatz. We recall that $\hat{E}_{p_3}$ provides an initial guess state with good overlaps on both the 1st and 4th excited subspaces: $F_2 \simeq 0.39$ and $F_4 \simeq 0.61$ respectively. For a WAVES variational search, as shown in fig. S4, a majority of times the final state collapses on the 4th excited subspace, and in the remaining on the 1st, always with high fidelity. This two-fold convergence averaged for 100 runs is reported in blue in fig. S8.

In order to implement FS, a value for the energy shift ϵ is required. A natural choice is to employ the energy of the initial guess state provided by $\hat{E}_{p_3}$ applied to the PH parametrized ground state: $|\Psi_3\rangle \equiv \hat{E}_{p_3}\hat{A}(\vec{\theta}_g)|\Phi\rangle$. Now, the energy of this trial state lies in between the two eigenvalues, corresponding to the excited subspaces with which we have consistent initial overlap. This makes them close in the folded spectrum and may become accidentally degenerate because of the initial uncertainty assigned to the set of parameters σ_{θ_g}. If it is chosen $\epsilon \simeq \langle|\Psi_3|\hat{\mathcal{H}}|\Psi_3\rangle$ in this way, the FS search in a minority of cases converges with high fidelity to the 4th excited subspace, but most of the time converges to local minima in the folded spectrum. These correspond to superpositions of eigenstates belonging to the different excited subspaces involved. In conclusion, the final excited states estimates are poor, though still resembling the two-fold behavior exhibited by WAVES (the results are shown in fig. S8, in red). These limitations in the adoption of FS are known from the literature, including the original work *(55, 56)*.

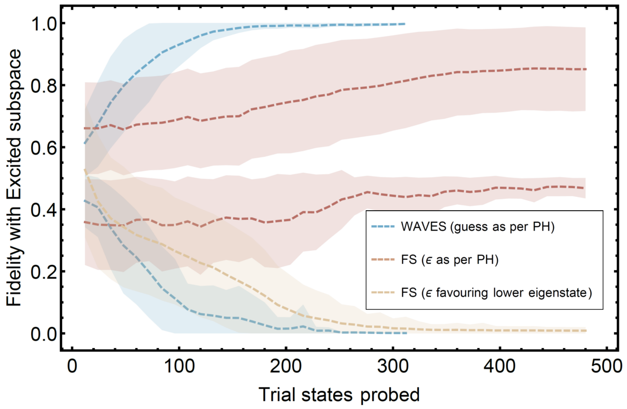

fig. S8. Behavior comparison of the first part of WAVES and an equivalent implementation of the FS method when applied to the initial guess provided by the $\hat{E}_{p_3}$ excitation operator for the H_2 system. FS simulations were run adopting two different values of the energy shift ϵ, color-coded as in the legend. The fidelity reported is with the 4th excited subspace. We remark how all cases for both WAVES and FS implementation that converge to states with $F_4 \simeq 0$ have $F_1 > 0.99$ and can be considered successful searches. In all simulations, binomial noise was included (see SM S 7).

In order to check the correct performance of the Folded Spectrum, we artificially reduced the value of ϵ to be slightly bigger than the eigenvalue of the 1st excited state. In this case, the FS search collapses consistently on the first excited subspace all the times, with a fidelity equivalent to that obtained with WAVES (see fig. S8, in yellow). It is still possible to observe a slowdown in the convergence, requiring about twice the number of trial states with an equivalent choice of parameters. This poorer convergence rate is an expected effect, because of the squaring of the condition number, due in turn to the squaring of the relevant operator $(\hat{\mathcal{H}} - \epsilon)^2$. The resulting cost may be even worse in a quantum implementation, where the classical matrix multiplications to compute folded energies are replaced by dimension-efficient squaring in the terms of the Hamiltonian. This tends to increase the cost of even a single energy evaluation by a polynomial amount on a quantum device.

This comparison highlights how a variational search implemented according to a FS method requires a careful choice in the energy shifts in order to produce reliable results. Obviously, whenever the energy spectrum is not known in advance, scanning different energies may be required to test the results obtained after a single run. For the same study case, WAVES variational search offers a solid alternative, successfully targeting excited eigenstates starting from poor initial guesses, with no a-priori knowledge of energy differences among the eigenstates.
